# Selection of Breast Biopsy Markers: Effect on Breast Imaging Procedures, Follow-up, and Costs

**DOI:** 10.1148/rg.250151

**Published:** 2026-08-13

**Authors:** Tanya W. Moseley, Beatriz E. Adrada, Elsa M. Arribas, Matthew W. Urban, Gina Hesley, Gary J. Whitman, Ronald A. Rauch, Mary S. Guirguis, Megha M. Kapoor, Christine U. Lee

**Affiliations:** ^1^Divisions of Radiology and Surgery, Departments of Breast Imaging and Breast Surgical Oncology, The University of Texas MD Anderson Cancer Center, 1515 Holcombe Blvd, Box 1340, Houston, TX 77030-4000; ^2^Division of Radiology, Department of Breast Imaging, The University of Texas MD Anderson Cancer Center, Houston, Tex; ^3^Division of Radiology Research, Department of Radiology, Mayo Clinic, Rochester, Minn; ^4^Division of Ultrasound, Department of Radiology, Mayo Clinic, Rochester, Minn; ^5^Division of Radiology, Departments of Breast Imaging and Breast Radiation Oncology, The University of Texas MD Anderson Cancer Center, Houston, Tex; ^6^Wesleyan University, Middletown, Conn; ^7^Division of Breast Imaging and Intervention, Department of Radiology, Mayo Clinic, Rochester, Minn

## Abstract

Breast biopsy markers have evolved from simple metallic markers to sophisticated multimaterial devices that serve critical purposes throughout the breast cancer care continuum. This evolution began at The University of Texas MD Anderson Cancer Center in the 1960s for gynecologic and head and neck tumors. After the MicroMark (Biopsys) clip was approved by the U.S. Food and Drug Administration in 1995, markers were subsequently adapted for patients with breast cancer in 1999. By 2003, markers had become the standard of care. Modern markers serve multiple clinical functions: marking biopsy sites, facilitating cross-modality imaging correlation, guiding surgical planning and radiation therapy targeting, and ensuring continuity of care across health care facilities. Long-term studies demonstrate excellent safety profiles with minimal adverse events and significant cost-effectiveness through improved surgical precision and reduced re-excision rates. Despite the widespread adoption of these markers, contemporary challenges persist, including marker migration, allergic reactions to metallic components or embedding materials, and visibility limitations on US images. However, recent technological advances address these concerns through improved marker designs, nonmetallic alternatives, and innovative detection methods. Current best practices emphasize optimal placement timing, appropriate marker selection based on patient-specific factors, migration prevention techniques, and use of Doppler US "twinkling" artifacts for enhanced US visualization.

^©^ The Author(s) 2026. Published by the Radiological Society of North America under a CC BY 4.0 license.

*Supplemental material is available for this article.*

## Graphical abstract

**Figure:**
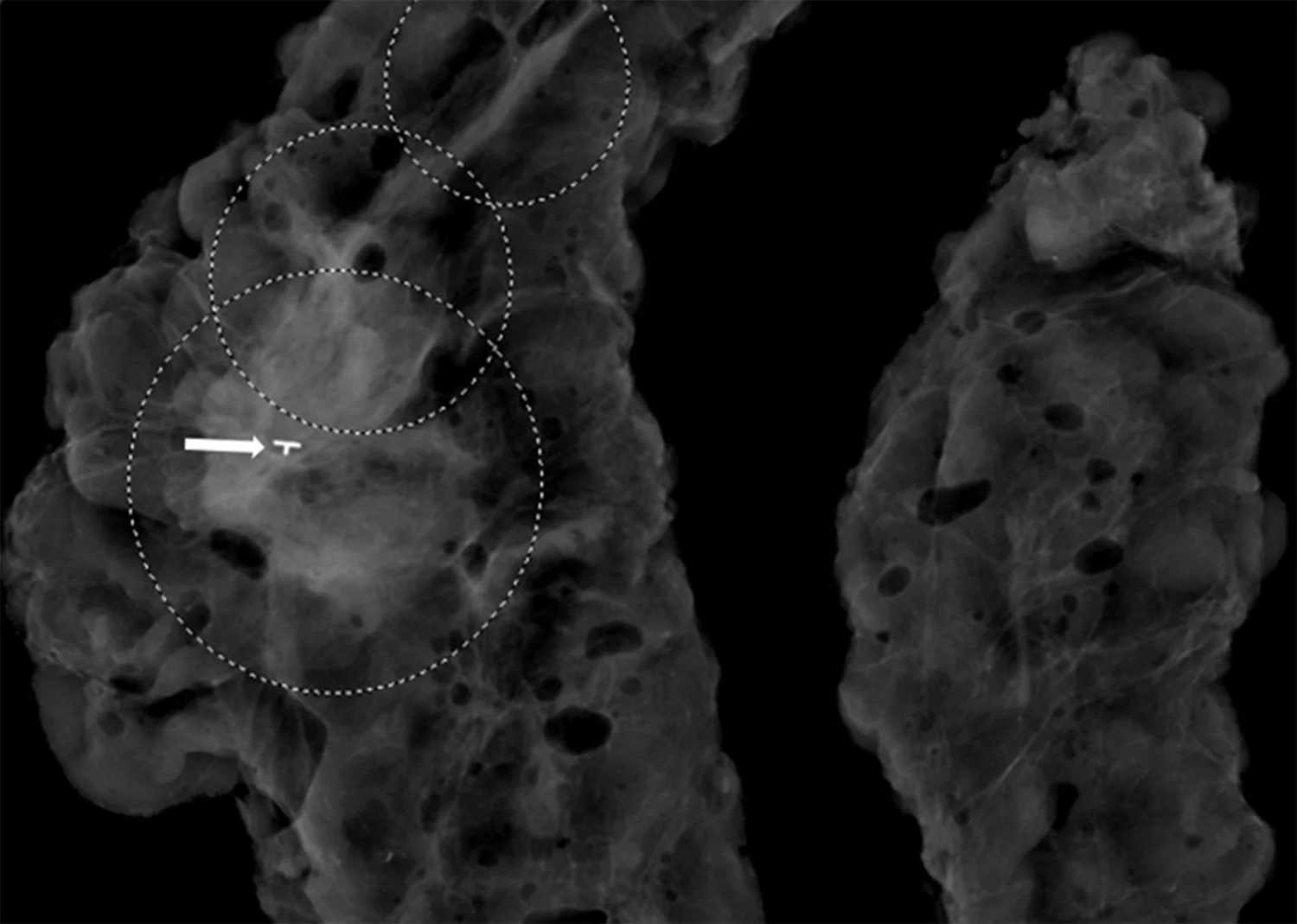

**Figure:**
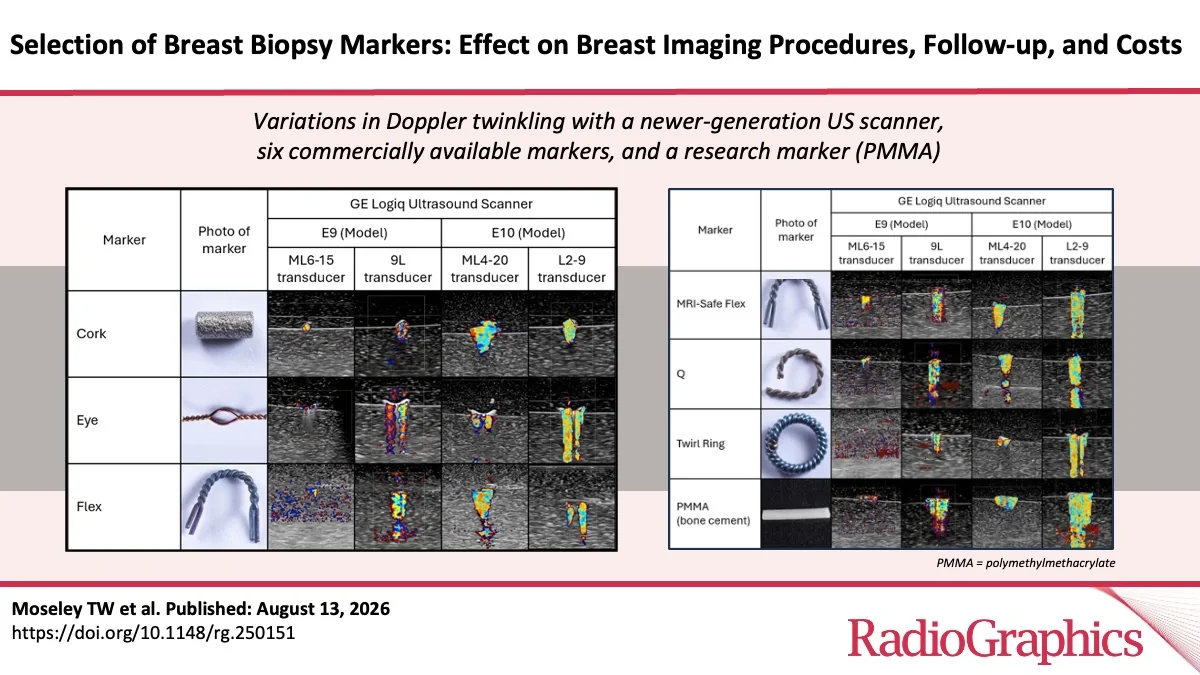

## Introduction

### History of Breast Biopsy Markers

The use of breast biopsy markers began in 1995, when the U.S. Food and Drug Administration (FDA) approved the first marker for implantation in soft tissue. The MicroMark clip (Biopsys Medical), featuring an innovative catheter-in-catheter design compatible with both vacuum-assisted probes and straight needles (1,2), was specifically designed to work in conjunction with a Mammotome (Mammotome) breast biopsy system, a vacuum-assisted device capable of complete removal of a lesion, with the potential to leave no identifiable landmark for future localizations.

### Evolution to Use in Clinical Practice

The transition from experimental use of breast biopsy markers to standard of care in clinical practice accelerated in 1999 when Edeiken et al (3) pioneered US-guided implantation of custom-made metallic markers in breast cancer tumor beds for patients receiving neoadjuvant chemotherapy before breast conservation surgery. This groundbreaking approach was adapted from techniques that were previously successful in gynecologic and head and neck tumors (4), allowing precise marking of tumor beds before complete chemotherapy response. The clinical significance became evident when 47% of patients showed metallic markers as the sole remaining evidence of the original malignancy at preoperative imaging (3).

By 2003, marker placement had evolved from an experimental procedure to standard practice for all stereotactic biopsies (5), subsequently extending to US-guided and MRI-guided biopsies. This transition marked a fundamental shift from early devices that functioned primarily as clips to modern markers serving as sophisticated fiducial markers for precise localization throughout the treatment continuum.

The aim of this comprehensive review is to discuss these markers, which have become essential to breast cancer care, demonstrating their clinical benefits in surgical planning, radiation targeting, and cost reduction while addressing contemporary challenges including marker migration, allergic reactions, and innovative detection methods such as Doppler US "twinkling."

## Clinical Benefits and Applications

The clinical utility of breast biopsy markers extends far beyond simple site marking. When they are deployed after biopsy, markers serve as permanent reference points that enable crucial clinical functions. Postbiopsy mammograms obtained after marker placement ensure accurate imaging correlation, while the markers themselves provide essential reference points in both breast and nodal procedures, enabling precise identification and tracking, regardless of pathologic results.

### Applications across the Disease Spectrum

The versatility of modern markers is apparent in different clinical scenarios. In patients with a benign breast lesion, markers facilitate systematic follow-up (6), allow surveillance of previous biopsy locations, and support medicolegal documentation requirements (1,3,5). For malignant or high-risk lesions, markers are even more critical, allowing identification of tumor bed sites for future reference and enabling precise targeting for subsequent interventions.

### Multimodality Imaging Integration

**Figure 1.**
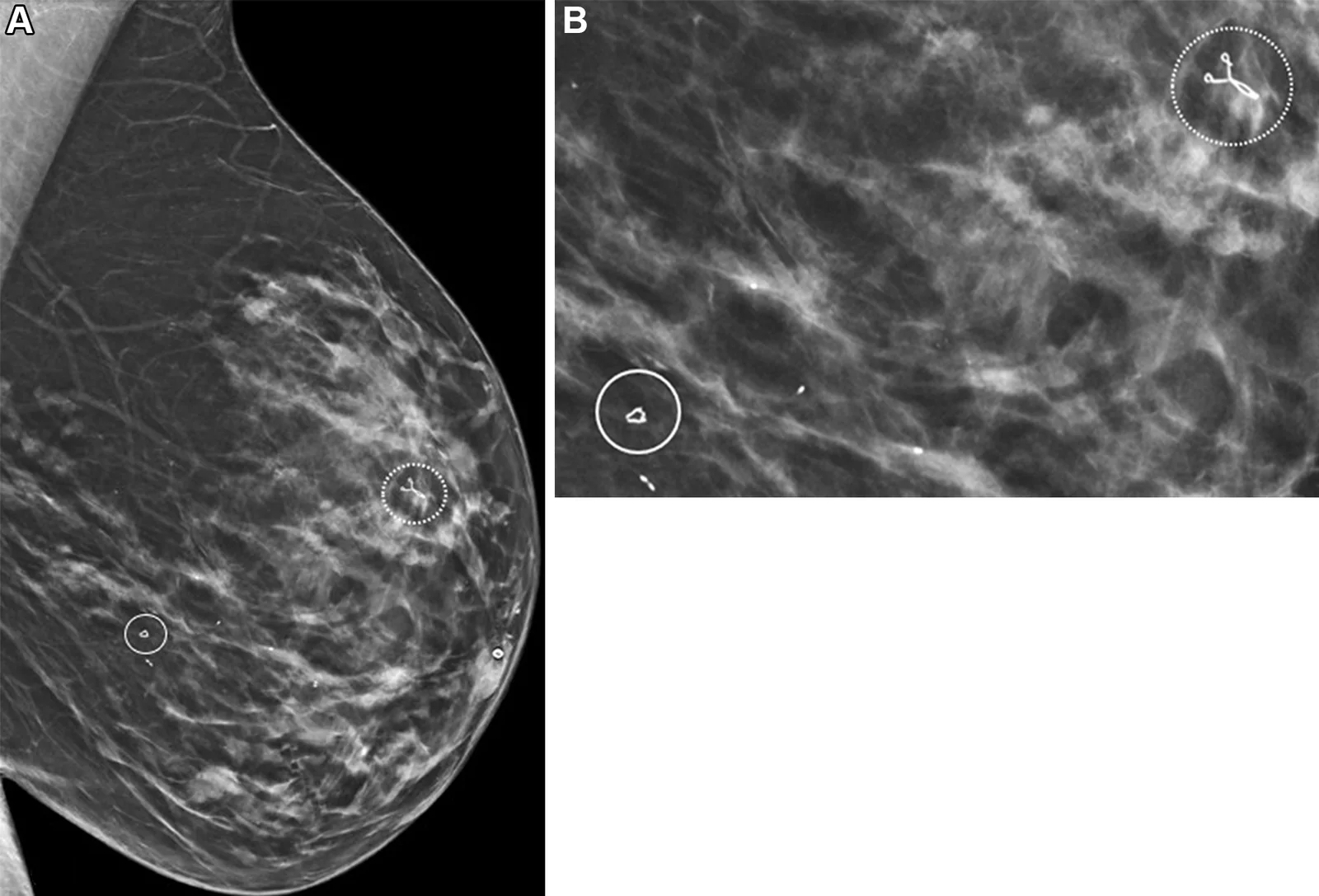
Differentiation of multiple biopsy sites using distinct marker shapes. Mediolateral mammograms in full-field **(A)** and magnified **(B)** views show an anchor-shaped marker (solid circle) and a dragonfly-shaped marker (dotted circle), each indicating the location of a prior biopsy that yielded benign results. The use of different marker configurations allows radiologists to distinguish between multiple biopsy sites within the same breast, facilitating accurate correlation during follow-up imaging and improving diagnostic precision.

Modern markers demonstrate remarkable versatility across imaging platforms, maintaining visibility on mammograms, US images, contrast-enhanced mammograms, and MR images. This cross-modality compatibility facilitates seamless imaging correlation throughout the patient’s care journey. The availability of markers in different shapes (Table, Table S1) further enhances their utility by enabling radiologists to distinguish multiple biopsy sites within the same breast (Fig 1), thereby improving diagnostic precision and reducing potential confusion during follow-up (7).

**Table:** Frequently Used Breast Biopsy Markers in Breast Imaging Practices

Marker Category and Type	Vendor	Brand	Common Names
US-guided			
Ribbon	BD	UltraClip	…
Professional Q	Hologic	Tumark	Q
Vision	Hologic	Tumark	…
Coil	BD	UltraCor	J-Coil
Ring	BD	UltraCor Twirl	…
Flex	Hologic	Tumark	U
Open Coil	Mammotome	HydroMARK	…
Butterfly	Mammotome	HydroMARK	…
O	BD	SenoMark	…
Stereotactically guided or tomosynthesis-guided			
Top Hat	Hologic	SecurMark	T
Cork	Hologic	TriMark	Rod, cylinder
Infinity	Hologic	SecurMark	…
Stoplight	Hologic	SecurMark	…
Hourglass	Hologic	TriMark	Dumbbell, barbell
Open coil	Mammotome	HydroMARK	…
Butterfly	Mammotome	HydroMARK	…
MRI-guided			
Hourglass	Hologic	TriMark	Dumbbell, barbell
Cork	Hologic	TriMark	Rod, cylinder
Spring	BD	UltraCor	Coil
Metal allergy			
Beacon	Mermaid	Mermaid	…
Barbell	Mammotome	MammoSTAR	Dumbbell

Note.—Markers are categorized by biopsy guidance method (ie, US-guided, stereotactic or tomosynthesis-guided, and MRI-guided) or by use in patients with metal allergies. Vendor names and brands are listed, with commonly used alternative names that may differ from manufacturer designations. The information presented does not constitute endorsement by the contributing authors or their institutions.

### Safety Profile and Clinical Efficacy

Extensive long-term studies consistently demonstrate the excellent safety profile of breast biopsy markers. Smith et al (8) conducted a comprehensive analysis of 768 markers, finding only three events (0.4%): two device deficiencies and one nonserious adverse event, with no serious adverse events reported. This remarkable safety record supports the widespread adoption of these devices in diverse patient populations.

Beyond safety, markers demonstrate exceptional accuracy and stability in clinical practice. Most markers remain within 1 cm of their target placement and exhibit minimal migration during follow-up periods. However, understanding the factors that can affect marker placement remains crucial for optimal outcomes. Suboptimal marker location typically relates to procedural factors, particularly excessive bleeding or hematoma formation, which can displace markers from the biopsy center. These complications occur more frequently with larger biopsies using vacuum-assisted systems with needles ranging from 12-gauge to 7-gauge, or when multiple biopsy passes are required (9).

### Enhancing Coordination of Patient Care

Modern health care delivery increasingly relies on seamless coordination of care across multiple facilities and providers. Breast biopsy markers are crucial to this coordination, providing definitive documentation of prior biopsy locations and effectively reducing the need for repeated biopsies when patients transition between providers (7). This capability proves particularly valuable for patients seeking second opinions or changing health care systems, ensuring continuity of care without compromising diagnostic accuracy.

### Optimizing Treatment Planning and Execution

**Figure 2.**
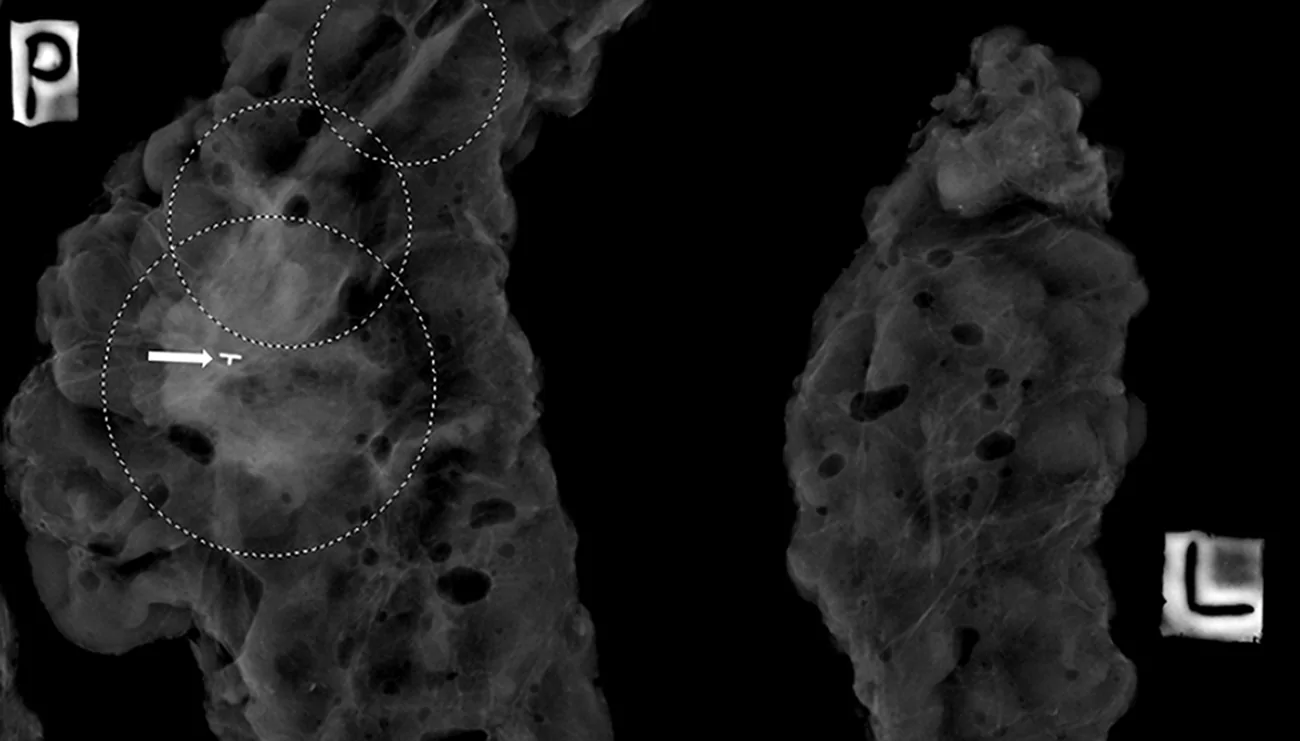
Postoperative radiograph of a specimen shows successful localization and excision of breast malignancy. Dashed circles outline the known malignancy in the excised tissue, with a radiopaque marker (arrow) used for preoperative localization. Specimen imaging provides immediate intraoperative confirmation of complete tumor excision and adequate surgical margins, allowing surgeons to assess excision adequacy before wound closure.

The integration of markers into treatment planning has revolutionized both surgical and oncologic approaches. In surgical planning, markers guide preoperative needle localization procedures with unprecedented precision, which is particularly valuable in patients presenting with multicentric and multifocal disease. Markers enable verification of accurate surgical excisions through specimen imaging, providing surgeons with immediate feedback on the completeness of excision (6) (Fig 2).

For cancer treatment, markers serve multiple critical functions. In patients receiving preoperative chemotherapy, markers provide a reliable means to monitor biopsy-proven malignancies, assess treatment efficacy, and guide subsequent surgical excision. In addition, markers define precise target areas for radiation therapy, enabling clinicians to minimize radiation exposure to surrounding healthy tissue while ensuring adequate coverage of the treatment area.

### Economic Benefits

The economic advantages of breast biopsy markers extend across multiple health care domains, creating compelling value propositions for health care systems. Improved surgical precision is the most substantial economic benefit, enabling accurate targeting of biopsy sites while reducing operative time and unnecessary tissue removal. This precision ensures complete lesion excision on the first attempt, allowing costly repeat procedures to be avoided (6, 10, 11).

A reduced need for repeat biopsies provides another substantial economic advantage. Permanent landmarks eliminate uncertainty about the location of the lesion during follow-up examinations, particularly when patients change providers or seek care at different facilities. Lower surgical re-excision rates result from precise preoperative localization, clear margins are achieved more consistently, and the substantial costs of repeat surgical procedures are avoided.

### Enhanced Patient Experience

From the patient perspective, markers provide tangible benefits that extend beyond clinical outcomes. Patients generally tolerate marker placement well, experiencing minimal discomfort during the procedure (12). More importantly, markers provide psychological reassurance of consistent monitoring and accurate tracking, substantially reducing patient anxiety.

The elimination of additional invasive procedures is perhaps the most important patient benefit. Markers enable reliable identification across all imaging modalities and facilitate smooth health care transitions, potentially reducing patient stress when seeking second opinions or changing providers (6,7,11). This continuity ensures that the biopsy history remains visually documented and readily accessible throughout the patient’s care journey.

## Contemporary Challenges and Solutions

### Nomenclature and Communication Issues

As the marker market continues to expand with new technologies and designs, standardized nomenclature becomes increasingly important for effective clinical communication. Current naming standards rely primarily on manufacturers’ designations, leading to variability across different practices and potential communication errors (13). While manufacturer-provided names are used in this article for clarity and consistency (Table S1), the field would benefit from standardized nomenclature to minimize errors and improve interprofessional communication.

### Marker Migration

Despite the general stability of markers, migration remains a substantial clinical challenge that can compromise the effectiveness of these devices. Understanding marker migration patterns and prevention strategies is essential for optimal clinical outcomes.

### Migration Timing and Mechanisms

Marker migration occurs during three distinct phases, each requiring different prevention strategies. Marker deployment migration happens when markers are deployed past the target or occurs after needle withdrawal due to tissue rebounding. Postprocedural migration occurs during compression for hemostasis or during postprocedural mammography when tissue manipulation displaces the marker. Follow-up imaging migration develops over time due to patient activities or target shrinkage during neoadjuvant therapy (14–17).

### Clinical Significance and Rates

Migration rates vary substantially across studies and techniques, ranging from 0%–38% of marker placements, depending on the definitions and the measurement methods used. Teichgraeber et al (17) reported concerning rates of 38% of markers migrating greater than 5 mm and 12% migrating greater than 20 mm, while Jain et al (18) documented 13% overall migration greater than 10 mm and 25.7% migration greater than 35 mm immediately after placement (16–22). These statistics underscore the importance of understanding contributing factors and implementing prevention strategies.

### Contributing Factors and Risk Assessment

Multiple factors influence migration risk, with breast thickness, global fatty breast density, and the biopsy approach emerging as primary contributors (19,22,23). The extent of biopsy-associated tissue changes appreciably affects migration risk, particularly when multiple samples are obtained with larger-gauge needles, creating substantial soft-tissue defects that predispose patients to marker displacement.

Migration patterns differ substantially across biopsy techniques. Stereotactic vacuum-assisted biopsies and MRI-guided procedures show higher migration rates due to the “accordion effect” during compression release (15–21,24–28). Conversely, US-guided marker placements performed without compression demonstrate markedly lower migration rates.

### Prevention Strategies

Effective prevention of migration requires attention to deployment techniques, device withdrawal, and postprocedural management. Slow controlled deployment allows real-time adjustments and minimizes the risk of propelling markers from the intended sites. However, markers with rapidly swelling embedding materials may require faster deployment to prevent device blockage.

**Figure 3.**
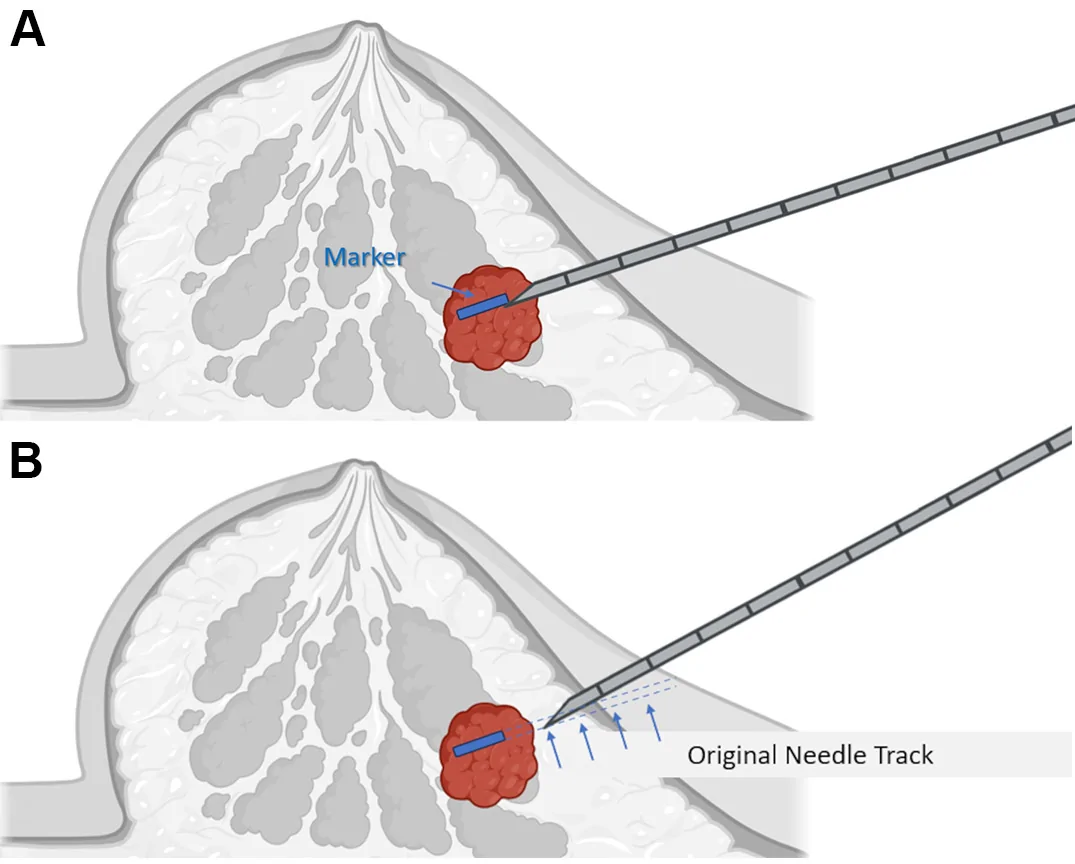
Changing the direction of needle withdrawal to minimize immediate marker migration. Illustrations show that once the marker is placed **(A)**, one can slightly change the direction of needle withdrawal **(B)**. Rather than creating a new track, this technique makes the least-resistive original needle track slightly more nonlinear to reduce the chances of the marker following the needle out when withdrawing the needle. (Created in BioRender, Urban M, 2025, https://BioRender.com/dno0i6n.)

**Figure 4.**
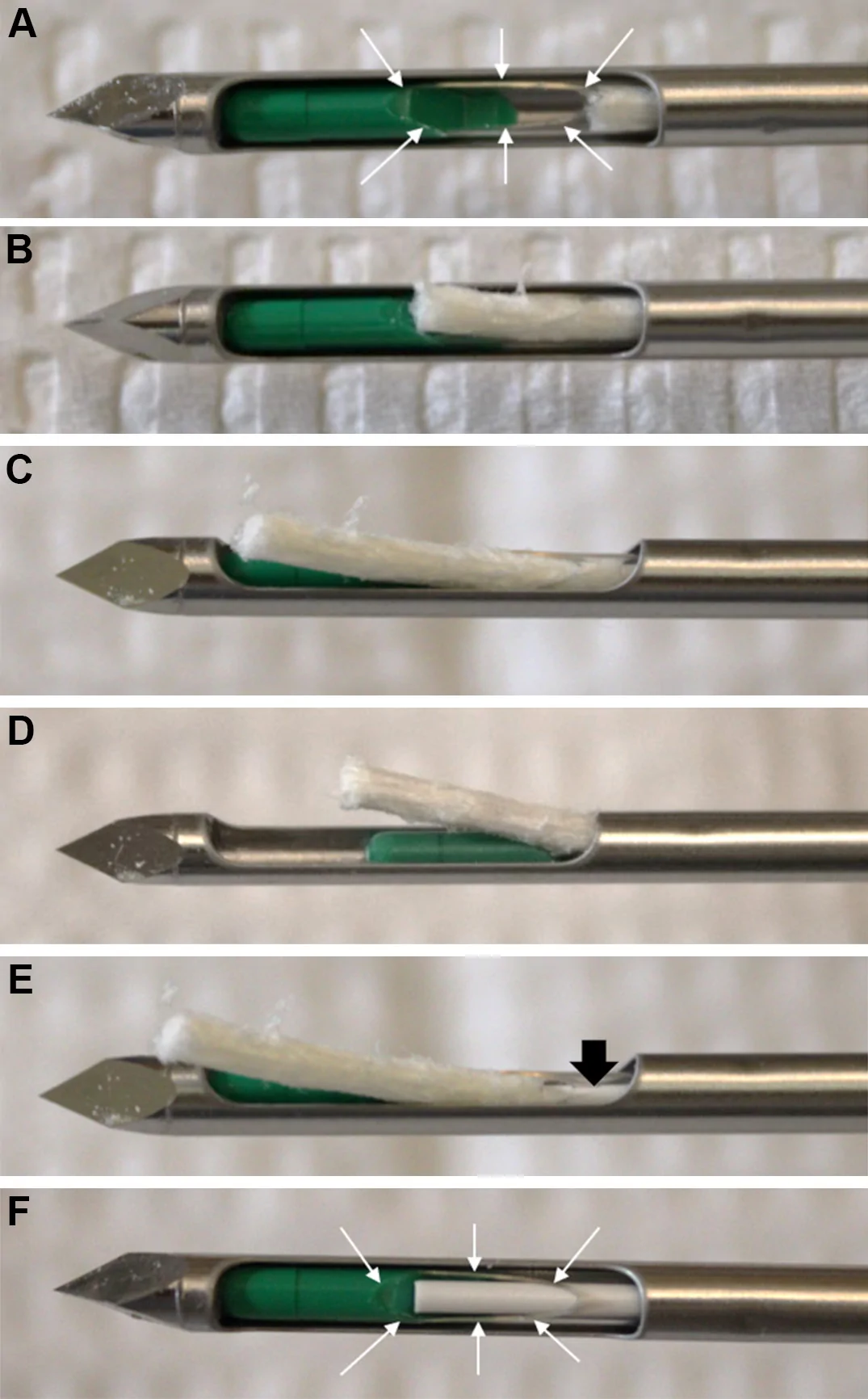
A technique to minimize immediate marker migration from a side-deployment marker deployment device. In this example, a bowtie marker is being deployed from its side-deployment device through the trough of a vacuum-assisted biopsy device. **(A)** On initial placement of the marker deployment device into the biopsy cannula, the trough of the marker deployment device (arrows) is lined up with the biopsy needle trough. **(B, C)** The bowtie marker with embedding material is slowly deployed from the deployment device. **(D)** If the device that pushes the marker out is released too soon, the marker may incompletely exit the troughs and get pulled back when the needle-marker system is withdrawn. **(E, F)** When deploying the marker, maintaining forward pressure on the pushing component (arrow in **E**) occupies the potential space of the marker deployment trough (arrows in** F**). By rotating the marker through so that the marker trough is 180° relative to the biopsy needle trough, the potential space for the marker or its embedding material to get caught is lessened.

During device withdrawal, specific techniques can minimize migration risk. Slightly angling the needle to create a different track or deploying markers with the bevel down is helpful in reducing suction effects (Fig 3). For side-deployment markers, rotating the deployment device 180° from the biopsy entrance site prevents catching of embedding material (29) (Fig 4).

Postprocedural management is focused on minimizing the accordion effect by slowly releasing compression during stereotactic and MRI-guided biopsies. Direct pressure over the biopsy site for hemostasis should help avoid excessive tissue manipulation, and repeated imaging should be considered when hematomas develop.

### Objective Assessment of Marker Migration across Imaging Modalities

Implementing a standardized quantitative assessment protocol can help address concerns regarding marker migration and cross-modality correlation in breast cancer management. With a standardized protocol, marker displacement is measured from the original biopsy site using consistent anatomic landmarks including the nipple-areolar complex, the chest wall interface, and the major vascular structures. Digital measurement tools integrated in picture archiving and communication systems (PACS) workstations can enhance precision and reduce operator variability.

Authors of various studies (19,22,23) have used different migration thresholds including greater than 10 mm (20,23), greater than 5 mm, and greater than or equal to 1 cm (19), highlighting the need for standardized thresholds. A tiered system incorporating minor (<5 mm), moderate (5–10 mm), and marked (>10 mm) displacement categories with associated clinical management recommendations may prove most useful.

For cross-modality follow-up imaging, marker position should be correlated, accounting for mammographic compression forces, MRI gravitational effects, and US probe pressure. Advanced image registration techniques can enhance correlation accuracy by automatically aligning anatomic structures.

Assessment of interobserver reliability should be targeted toward substantial agreement (κ > 0.61) based on the Landis and Koch 1977 guidelines (30), with training protocols established throughout multiple observer categories including radiologists and breast surgical oncologists. Such methods allow quantification of migration rates across imaging modalities and provide objective data on marker visibility for surgical targeting (30).

### Postprocedural Patient Mobility Guidelines

A substantial limitation in current breast biopsy practice is the lack of standardized guidelines for postprocedural mobility among institutions, which may contribute to variable marker migration rates. The authors’ review of current protocols from major medical institutions reveals considerable inconsistency in recommendations for activity, with physicians from some centers allowing immediate return to normal activities, while others restrict strenuous activity for several days. Most institutions provide general recommendations to avoid strenuous activity for 24–48 hours, but specific guidance regarding arm and shoulder movements that might influence the position of markers is inconsistently addressed. This variability may be an unrecognized factor contributing to marker migration. The authors recommend that institutions consider developing specific protocols for postprocedural mobility including three key components detailed in the following sections.

***Immediate Restrictions on Ipsilateral Arm Elevation for 2–4 Hours.—***The protocol during this critical initial period is focused on preventing acute displacement of markers during primary hemostasis and early formation of clots. Patients should maintain the ipsilateral arm below shoulder level, avoiding reaching overhead, washing hair, dressing that requires arm elevation, or placing objects on high surfaces. The arm remains functional for activities at or below shoulder height, including eating, writing, and light computer work. This restriction specifically targets prevention of contraction of the pectoralis muscle and stretching of the breast tissue that occurs with overhead movements, which could theoretically disrupt the newly placed marker before initial tissue stabilization occurs.

***Graduated Activity Recommendations with Specific Timelines.—***A structured progression should guide patients through phases of increasing activity. During hours 4–24 after the procedure, a gradual increase in shoulder-level movements is recommended, while maintaining overhead restrictions and avoiding sudden or forceful motions. During the 24–72-hour phase, a return to most normal activities is permitted, except for heavy lifting (>10 or >15 lbs, depending on the patient’s condition) and vigorous exercise. After 72 hours, nearly all activities can be resumed, with only high-impact sports or intensive upper-body exercise restricted until a clinical follow-up examination. This timeline respects the biologic healing process while providing clear actionable guidance that is clearer than vague instructions to "take it easy."

**Figure 5.**
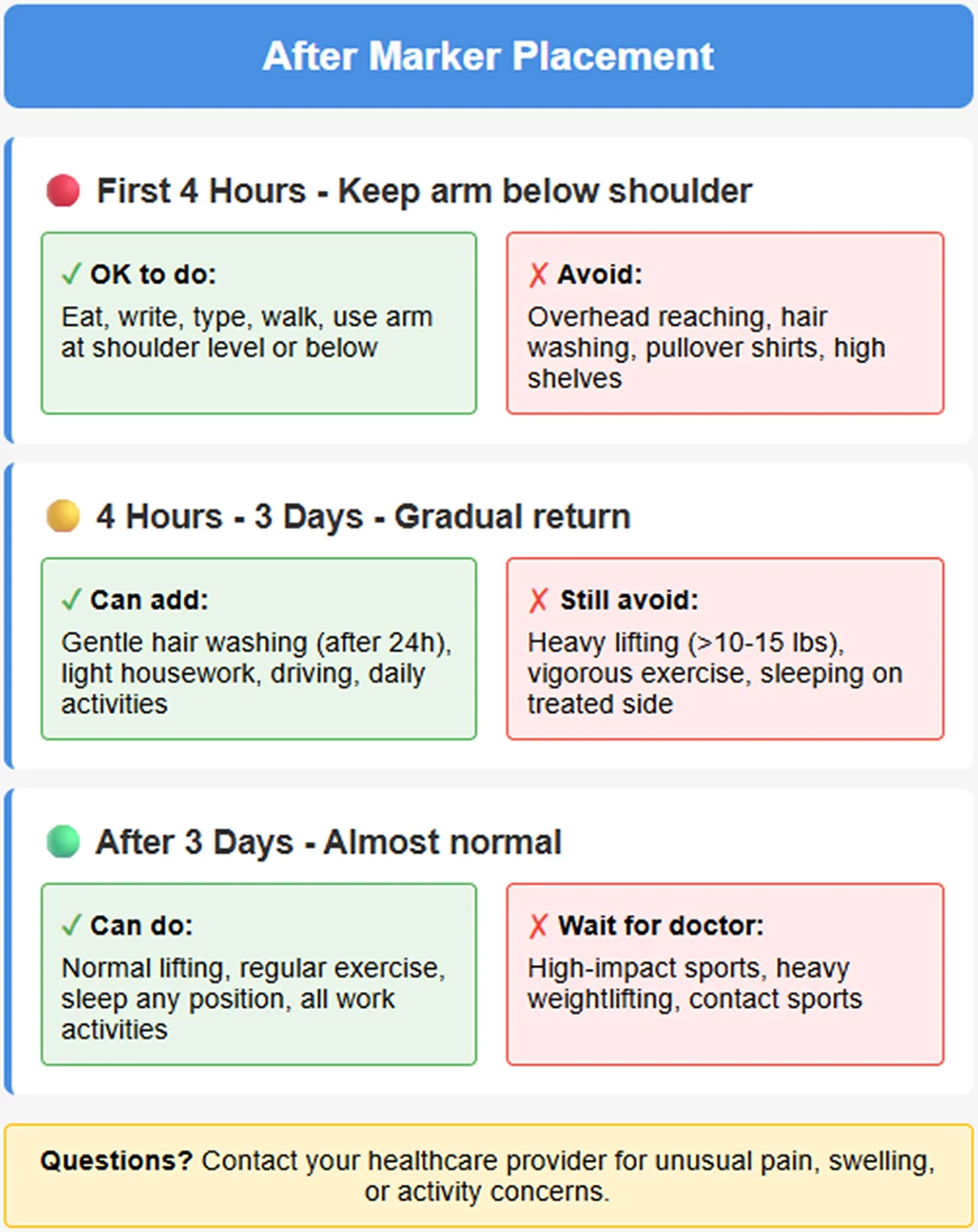
Patient education guide shows activity restrictions after placement of markers. The three-phase recovery timeline shows evidence-based activity restrictions designed to prevent marker displacement during the critical initial healing period. This structured approach replaces vague instructions to "take it easy" with specific timeline-based guidance that respects biologic healing processes while optimizing patient adherence through demonstration of the rationale for the restrictions.

***Patient Education Regarding Activities That May Affect Marker Stability.—***Comprehensive education should address specific high-risk activities including sleeping positions (eg, avoiding the ipsilateral side for 24–48 hours); occupational demands (eg, workplace accommodations for jobs requiring overhead reaching); resumption of exercise (eg, walking immediately, cycling after 48–72 hours, and high-impact activities after 5–7 days); and daily activities such as driving, housework, and personal care routines. Education and successful implementation must emphasize the rationale behind restrictions to improve adherence and should include visual aids demonstrating permitted and restricted movements at each phase. (Fig 5). Future researchers should investigate the relationship between specific activities and marker migration rates, potentially using accelerometer data to develop evidence-based protocols that balance marker stability with patient quality of life.

### Allergic Reactions: Recognition and Management

Although rare, allergic reactions to breast biopsy markers are important clinical considerations, particularly given the increasing diversity of marker materials and embedding components. To our knowledge, only scattered case reports of allergic reactions to breast markers appear in the literature and the FDA Manufacturer and User Facility Device Experience (MAUDE) database (31–34). However, the potential for reactions requires careful consideration of both metallic components and embedding materials.

***Metal Allergy.—***Nickel is the most common metal allergen (affecting 11.4% of the population), with women having higher sensitivity rates (15.7%) than men (4.3%)(35–38). Cobalt and chromium sensitivities follow, at 2.7% and 1.8%, respectively (35).

Modern marker design addresses these concerns through careful material selection. Nitinol markers, despite containing nearly equal parts nickel and titanium, remain popular due to their superior shape-memory properties. Different stainless-steel grades offer varying nickel content, with 316 L steel containing 10%–14% nickel, while BioDur 108 (Carpenter Technology), which is used in the SenoMark coil (Beckton Dickinson [BD]) and Venus markers (BD), contains only 0.1% nickel. Titanium markers have gained popularity due to their virtual absence of nickel, with reaction rates as low as 0.6% (39,40).

For patients with metal allergies or situations in which metal artifacts could compromise imaging quality, nonmetallic radiopaque alternatives provide viable options (6). These include carbon-coated zirconium oxide markers (Barbell and Tribell) and polyetherketoneketone (PEKK) markers (Beacon and Cassi Star; Mermaid Medical), offering excellent imaging characteristics without metallic components.

***Embedding Material Sensitivities.—***Embedding materials present additional allergy considerations, as they may be the primary tissue contact point, depending on marker design. Common embedding materials include polyethylene glycol (PEG), collagen, bioabsorbable suture-like netting, β-glucan, polyvinyl alcohol, polyglycolic acid, and starch pellets. Screening for sensitivities to these materials should accompany the evaluation for allergies to the metallic components of markers.

**Figure 6.**
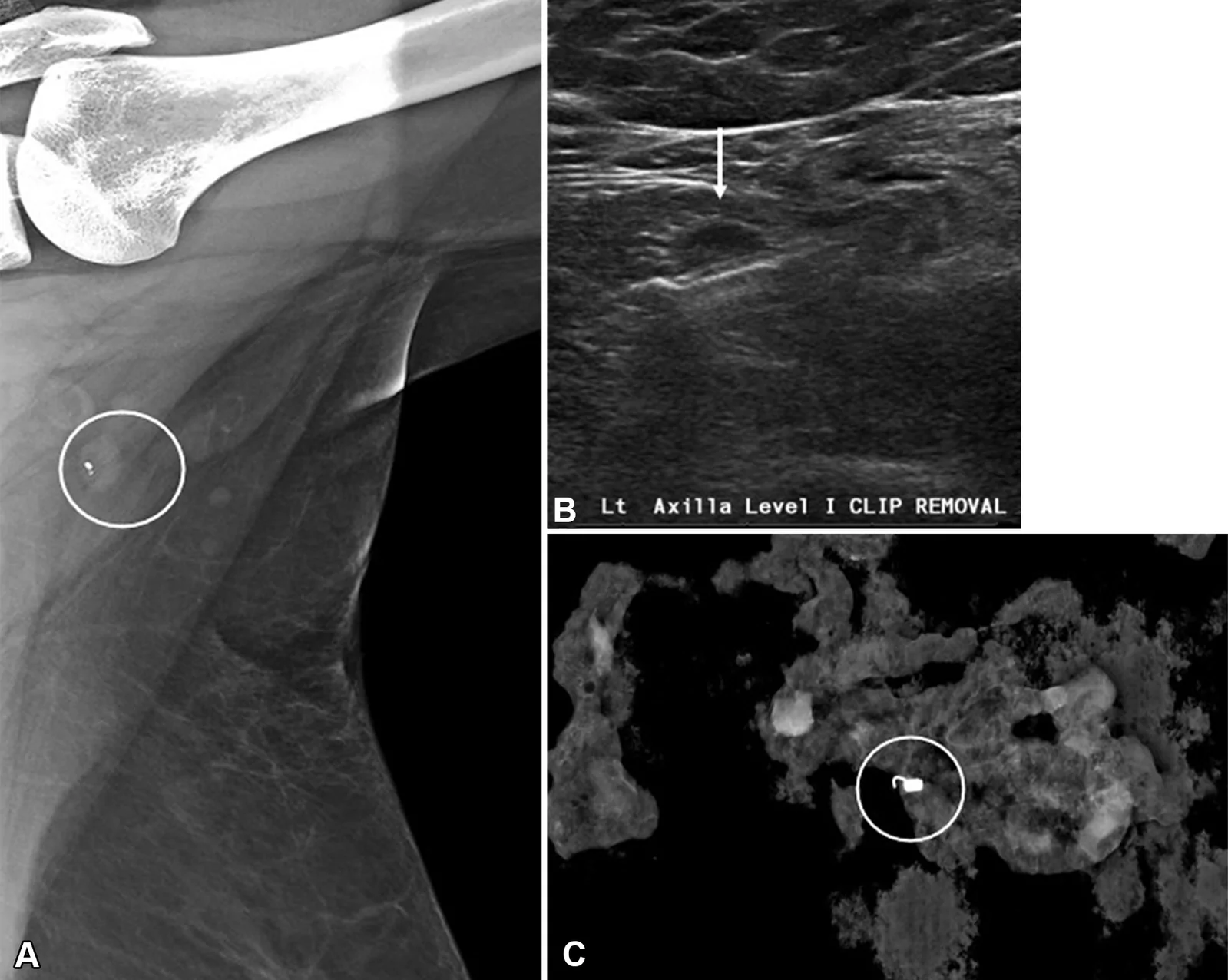
Allergic reaction to breast biopsy marker requiring percutaneous removal in a 43-year-old woman with left axillary pain and unusual body odor after US-guided lymph node biopsy and marker placement. The patient had no history of body odor despite an active lifestyle. **(A)** Left mediolateral oblique mammogram shows an axillary level 1 lymph node containing a radiopaque marker (circle). **(B)** US image shows vacuum-assisted percutaneous removal of the lymph node and marker (arrow). **(C)** Specimen radiograph allows confirmation of complete removal of the marker (circle). The patient’s pain and body odor completely resolved after marker removal, confirming allergic reaction as the underlying cause. This case illustrates the importance of recognizing atypical presentations of marker allergy and the effectiveness of percutaneous removal when conservative treatment fails.

***Clinical Recognition and Treatment.—***Allergic reactions typically manifest as skin erythema, rashes, pain, and pruritus (41–43). Postbiopsy erythema may initially be attributed to procedural inflammation or infection, but persistent symptoms that fail to resolve with conservative treatment or antibiotics should raise suspicion for allergic reactions. Treatment with antihistamines and steroids generally provides effective symptom relief (Fig 6).

## Marker Extraction: Techniques and Indications

The ability to remove markers when clinically indicated is an important aspect of comprehensive marker management, requiring specialized techniques and careful patient selection.

Percutaneous marker extraction involves the use of vacuum-assisted biopsy devices targeting the marker of interest. Stereotactic guidance remains the most successful approach, with large-gauge (ie, 11 gauge or larger) vacuum-assisted systems providing optimal marker removal while preventing entanglement in the needle trough (44,45).

US-guided extraction has emerged as an alternative approach using large-gauge needles such as the EnCor Enspire (BD) 7-gauge system (46). However, marker type substantially influences extraction success, with larger markers such as those with a ring configuration (UltraCor or Twirl; BD) prone to entanglement and migration during vacuum-assisted procedures (47, 48).

The primary indication for marker extraction involves retained markers after surgical excision of breast cancer. Retained markers after breast cancer surgery warrant extraction and tissue sampling, as 39% harbor residual malignancy (49), making extraction and tissue sampling a crucial diagnostic tool. Additional indications include documented allergic reactions to markers and the patient’s emotional distress related to the presence of a foreign body (44).

## Optimizing Marker Selection and Placement

Effective marker selection requires systematic consideration of the clinical intent, patient factors, and anticipated treatment pathways. The fundamental question "Why am I placing this marker?" should guide every placement decision, directly informing the selection of the marker type and the placement strategy. Whether markers are used to mark a biopsy-proven malignancy, delineate the extent of disease, or facilitate cross-modality correlation, marker visibility, particularly on US images, must be a primary consideration.

### US Visibility: Challenges and Solutions

While markers show excellent conspicuity on mammograms, US visibility presents ongoing challenges that substantially affect clinical utility. US detection depends on multiple factors including operator training, equipment variability, and marker-specific characteristics.

***Embedding Materials and Visibility Enhancement.—***Embedding materials serve dual purposes: improving marker stability and enhancing US conspicuity. Polyglycolic acid microfiber pads extend approximately 5 cm when deployed, creating substantial space-occupying effects. Many embedding materials swell on contact with biopsy-associated fluids, including hydrogel containing polyethylene glycol, β-glucan, and polyglycolic acid microfiber components.

***Hydrogel Technology.—***HydroMARK (Mammotome) markers represent sophisticated attempts to optimize US visibility through hydrogel expansion up to 200% of the original size within 24 hours. This expansion persists for up to 12 months at US while maintaining mammographic and MRI visibility through the metallic components. However, challenges include progressive gel dissolution over time and operational difficulties, with intraoperative complications reported in more than 50% of cases due to hydrogel slickness and extrusion tendencies (50,51).

***Advanced Form Factors.—***Modern marker design emphasizes enhanced US visibility through increased size (from 1–3 mm in early designs to 8–9 mm in contemporary versions) and sophisticated configurations using nitinol shape-memory properties. UltraCor Twirl markers (BD) and Tumark Vision (Hologic) markers exemplify this approach, offering three-dimensional structures with enhanced echogenicity specifically designed for detection at US.

Despite these technological advances, breast biopsy markers remain invisible at US up to 50% of the time, particularly after neoadjuvant therapy and in axillary applications. This limitation underscores the importance of alternative detection methods and careful marker selection.

**Figure 7.**
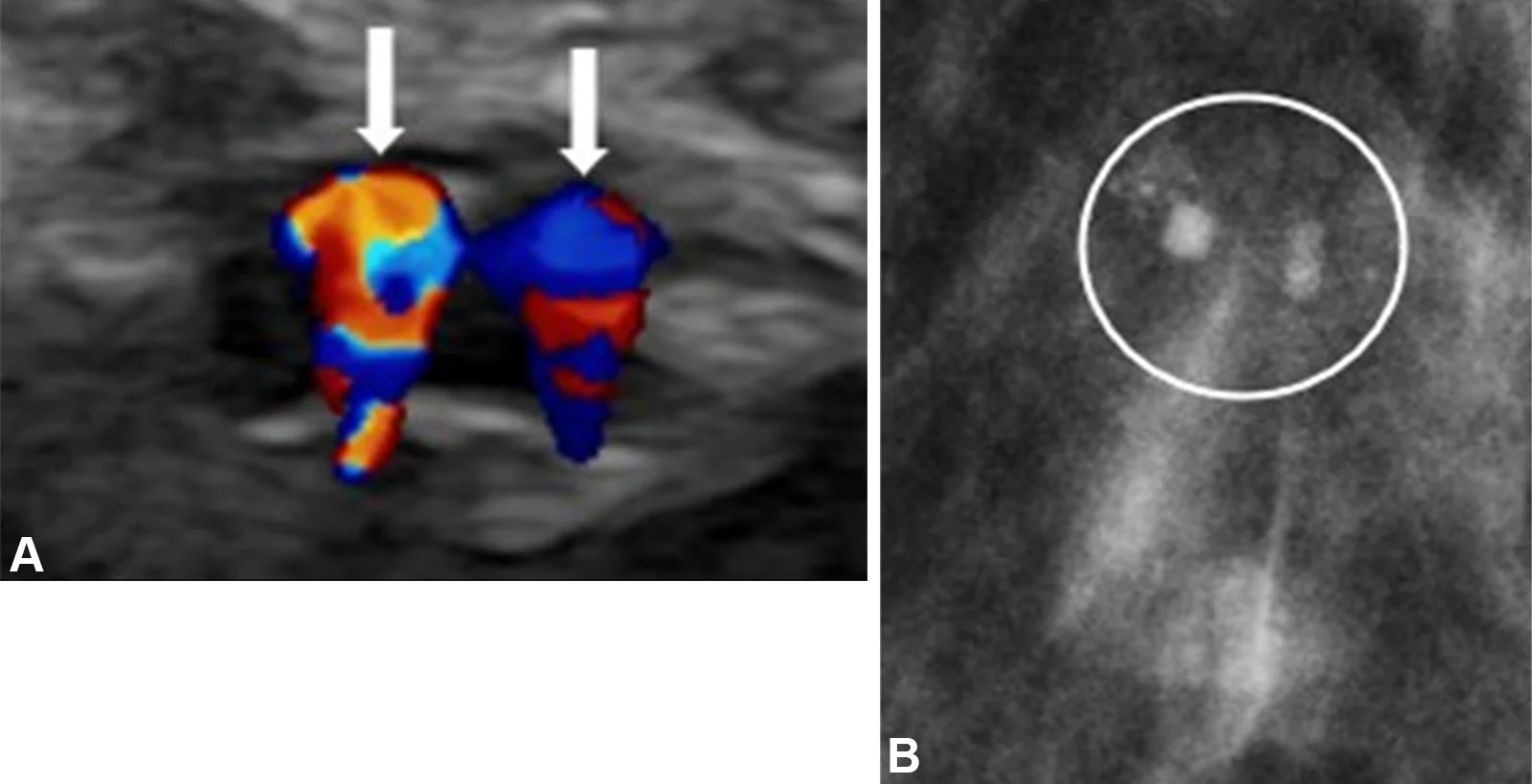
Twinkling artifact of breast calcifications. **(A)** Color Doppler US image shows twinkling artifacts (arrows). **(B)** Electronically magnified mammogram shows the correlating calcifications (circle). Twinkling artifacts, which were originally described for detecting reflective entities such as urinary stones, can be seen in patients with breast calcifications.

***Color Doppler US Twinkling: Revolutionary Detection Method.—***The application of color Doppler US twinkling artifacts to detection of breast markers is a substantial technological advancement. Originally described in 1996 by Rahmouni et al (52) for detection of reflective entities such as urinary stones, twinkling appears as rapidly changing colors deep to strongly reflective surfaces during color Doppler US examination (Fig 7).

**Figure 8.**
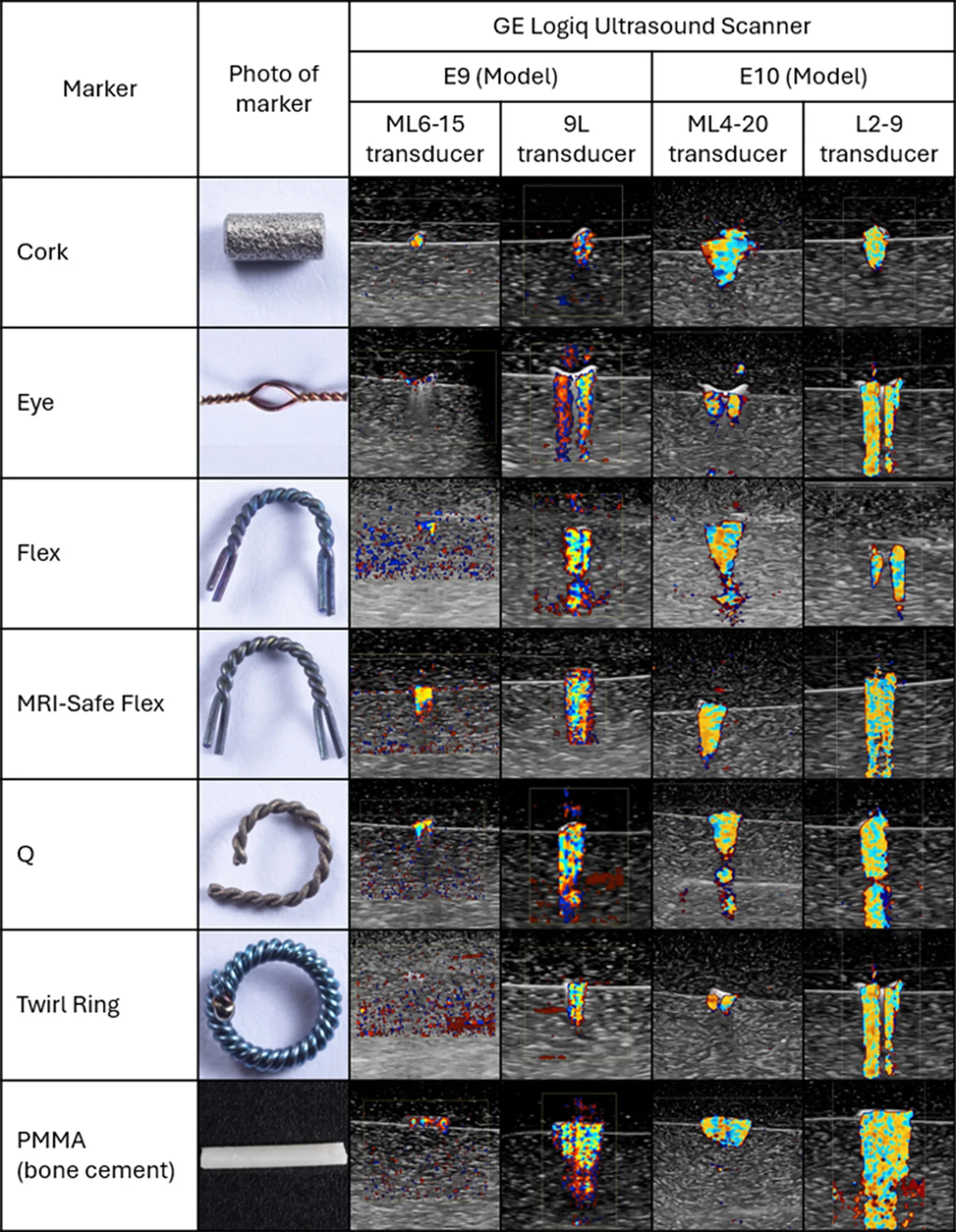
Chart shows slight variations in Doppler twinkling with a newer-generation US scanner, six commercially available markers, and a research marker made of polymethylmethacrylate *(PMMA)*.

**Figure 9.**
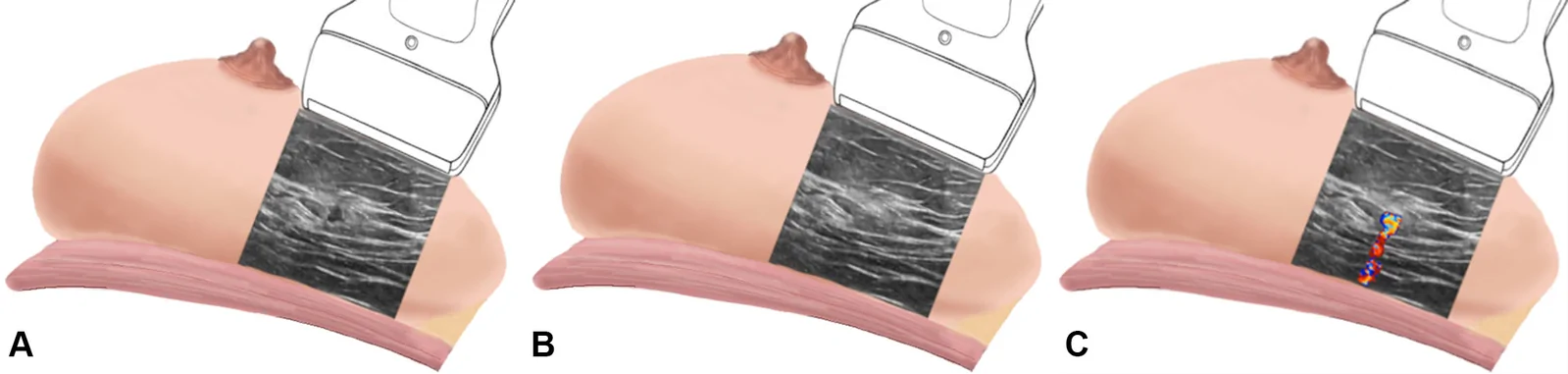
Color Doppler twinkling for marker detection. **(A, B)** Illustrations show that a biopsy-proven breast cancer responds favorably to neoadjuvant systemic therapy **(A)**, but the marker is challenging to identify at B-mode US **(B)**. **(C)** Some markers show twinkling at color Doppler US.

Specific markers demonstrate reliable twinkling characteristics, including Professional Q (Hologic), Cork (Hologic), MRI Flex (Somatex), and Ring (BD) configurations in certain orientations (53–55) (Fig 8). Authors of clinical studies (53,54) have demonstrated remarkable success rates, with all markers successfully identified using twinkling in cases in which they were unidentified with conventional B-mode US (56,57) (Fig 9). Color Doppler US twinkling can help identify 100% of compatible markers when conventional B-mode US is unsuccessful and is a vendor-agnostic technique requiring minimal adjustment of parameters, investment of time, and training. Optimal detection of twinkling involves initial scout imaging sweeps using lower-frequency transducers (3–6 MHz linear or 2–4 MHz curvilinear arrays), followed by higher-frequency imaging for detailed characterization. This approach provides cost-effective detection of markers without additional equipment requirements (53,54,58).

### Anatomically Challenging Locations

Far medial, posterior, and lateral lesions in anatomically challenging locations require specific marker selection strategies to ensure successful localization throughout treatment. For positions that are difficult to target with conventional mammographic guidance, US-visible markers are essential, particularly when neoadjuvant therapy is anticipated (53,54).

Management of superficial lesions requires careful balance between marker visibility and patient comfort. Bare markers (ie, breast markers made of metal only) offer smaller profiles that reduce palpability concerns when placed superficially, but most of these markers are small and lack adequate US visibility. Exceptions include the Cork (Hologic), which is relatively small and can be detected with twinkling, or the Open Coil, Barrel, and Butterfly (all from Mammotome), which are small and are US visible because of the hydrogel embedding material.

### Lymph Node Applications

**Figure 10.**
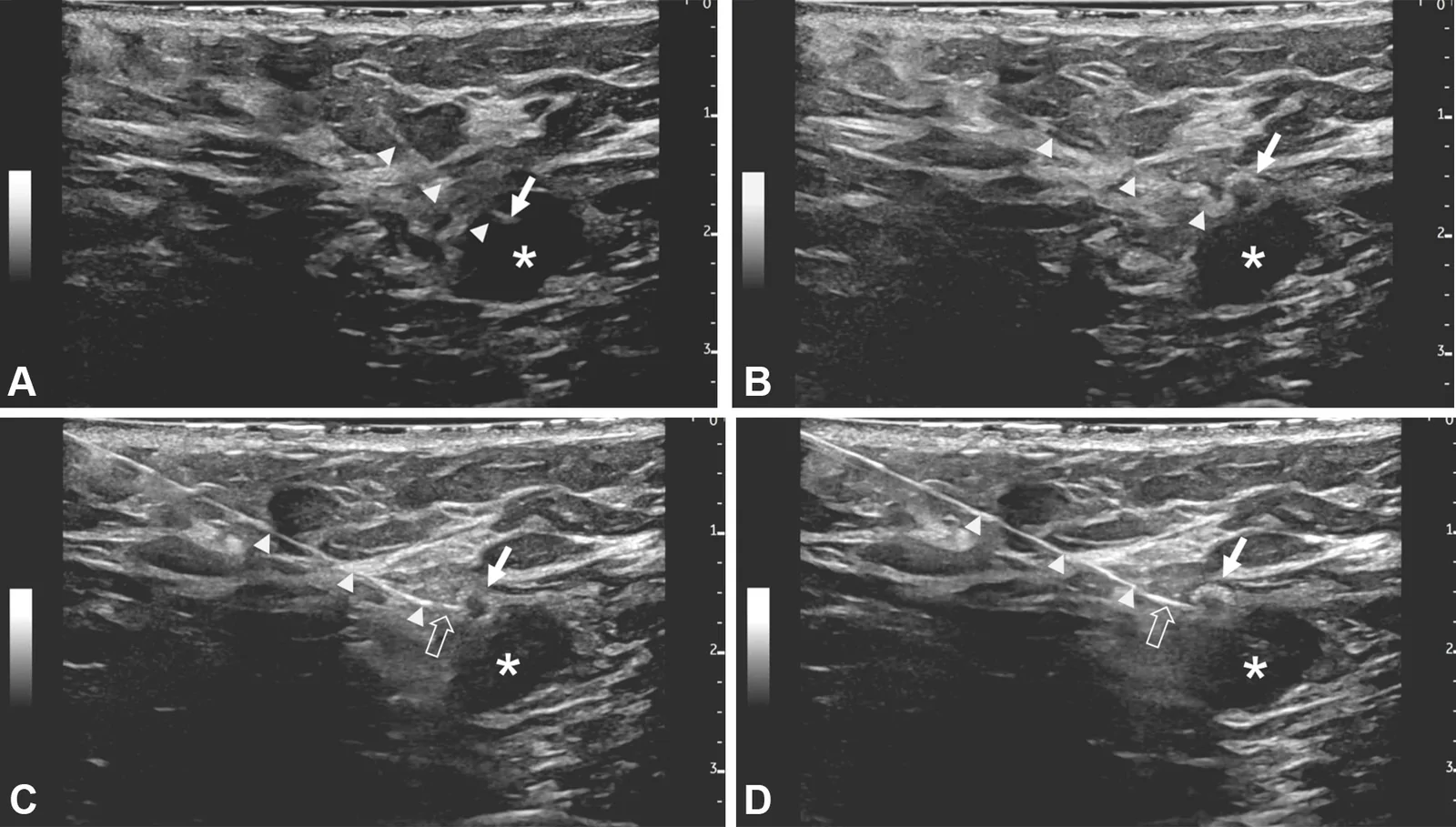
Misplacement of a Q marker due to marker expansion and immediate displacement. US images show a deployment needle at the outer portion of the cortex of an axillary lymph node (*). The deployment needle (arrowheads in** A–D**) is positioned at the outer portion of the lymph node cortex. Since the marker is elongated and without a sharp tip, it tends to push away the lymph node during deployment and expansion. As the Q marker slowly exits the beveled tip of the deployment needle (solid arrow in **A**), formation of the nitinol marker into a Q shape expands the marker, causing it to be immediately adjacent to the lymph node (solid arrow in **B**). Initial withdrawal of the needle introduces air into the tract (open arrow in** C**). The suction created on withdrawal of the needle tends to pull the Q marker (solid arrow in** D**) along with it. Air can be seen in the needle track as it is withdrawn (open arrow in **D**). To avoid misplacement, the marker deployment needle should be placed more centrally and deeper in the cortex of the lymph node.

Axillary lymph node marking presents specific challenges requiring specialized approaches. US is the preferred modality for visibility and is critical for successful outcomes. Placement in the hypoechoic cortex rather than in the hyperechoic fatty hilum substantially enhances detection capabilities (Fig 10). Clinical outcome studies (14,59) have provided compelling evidence for marker selection. This is particularly true in the axilla when localization is unsuccessful due to poor US conspicuity of the marker up to 25% of the time (14,59) and even 50% of the time with the Tumark (Hologic) and HydroMARK (Mammotome) markers (14,59). The Twirl (BD) marker showed 83.3% detectability in nodes without residual abnormality compared with 42% detectability for UltraClip (BD) markers (60). These findings emphasize the critical importance of marker selection for lymph node applications. For axillary lymph node marking, place markers within the hypoechoic cortex rather than in the hyperechoic fatty hilum to enhance US detectability. In axillary lymph nodes, Open Coil (Mammotome) markers have the highest localization success rate (85%), followed by Professional X (Hologic) (76%) and Flex (Somatex) (68%) markers, significantly outperforming UltraClip (BD) markers (42%).

### Neoadjuvant Therapy Considerations

Neoadjuvant therapy applications require markers capable of maintaining visibility throughout extended treatment periods, typically spanning 6 months. Reliable long-term marker performance remains essential to support accurate localization throughout the continuum of breast cancer care. Multiple marker systems (Table S1) address these demanding requirements, including the Hologic expandable nitinol series (Flex, Professional Q, Professional X, Vision, Eye, and Conic), which provide structural expansion after deployment, with enhanced stability and echogenicity; UltraCor Twirl markers, which offer expandable nitinol construction with proven long-term visibility; Mammotome HydroMARK systems (Open Coil, Barrel, Butterfly), which maintain US visibility for 11–15 months through hydrogel technology; nonmetallic or carbon-based markers (Barbell, Tribell), which use expanding polysaccharides for sustained visibility; and Beacon and Cassi Star markers, which involve the use of barium sulfate for permanent US visualization.

### Cross-Modality Correlation

Complex cases requiring correlation across multiple imaging modalities benefit greatly from strategic placement of markers. When mammographic lesions have potential US correlates, markers help confirm correct targeting and facilitate future correlation. Reviewing prior imaging is essential to avoid confusion, with different shapes selected when existing markers are present (61).

MRI applications require particular attention to marker composition and artifact characteristics. Stainless steel and BioDur markers produce prominent susceptibility artifacts, while titanium alloys generate intermediate effects. Carbon-coated ceramic and PEKK markers minimize signal intensity void artifacts, preserving tissue visualization capabilities. Understanding these characteristics enables optimal selection of markers for specific imaging requirements. For MRI applications, carbon-based ceramic and PEKK markers produce minimal artifacts compared with stainless steel markers, which create prominent susceptibility artifacts that may obscure tissue evaluation.

### Nonwire Localizers for Surgical Localization: Visibility and Clinical Applications

Building on traditional biopsy marker technology, nonwire localizers have revolutionized breast surgical localization by offering major advantages over traditional wire-guided techniques, including flexible scheduling, improved patient comfort, and the ability to place devices days to weeks before surgery. The three primary systems in clinical use in the United States are Savi Scout (Merit Medical) radar localizers, Magseed magnetic localizers (Endomag, Hologic), and localizer radiofrequency identification (RFID) tags (Hologic). An important clinical evolution involves using these nonwire localizers as dual-purpose devices, serving both as biopsy markers and surgical localization systems to streamline patient care. However, clinical experience suggests that predicting the appropriateness of dual-purpose device placement at the time of biopsy can be challenging. In one small institutional study (62) of MRI-guided procedures, 60% (6/10) of Scout devices placed at the time of MRI-guided biopsy were not subsequently used for localization surgery, highlighting the importance of developing institutional protocols for patient selection when implementing dual-purpose localization strategies. Similar studies examining this clinical question for Magseed and localizer systems are lacking, representing an important gap in the literature that warrants future investigation as institutions increasingly adopt dual-purpose placement strategies.

The visibility characteristics of nonwire localizers lend themselves to clinical implications for patient selection and follow-up care. At mammography, all three systems are radiopaque and demonstrate US visibility. While a study by Cario et al (63) demonstrated that the US viewing angle can greatly affect clip conspicuity and that morphologic changes during treatment may impact the visibility of biopsy markers, similar challenges are expected to affect nonwire localizers given their reliance on US detection principles.

**Figure 11.**
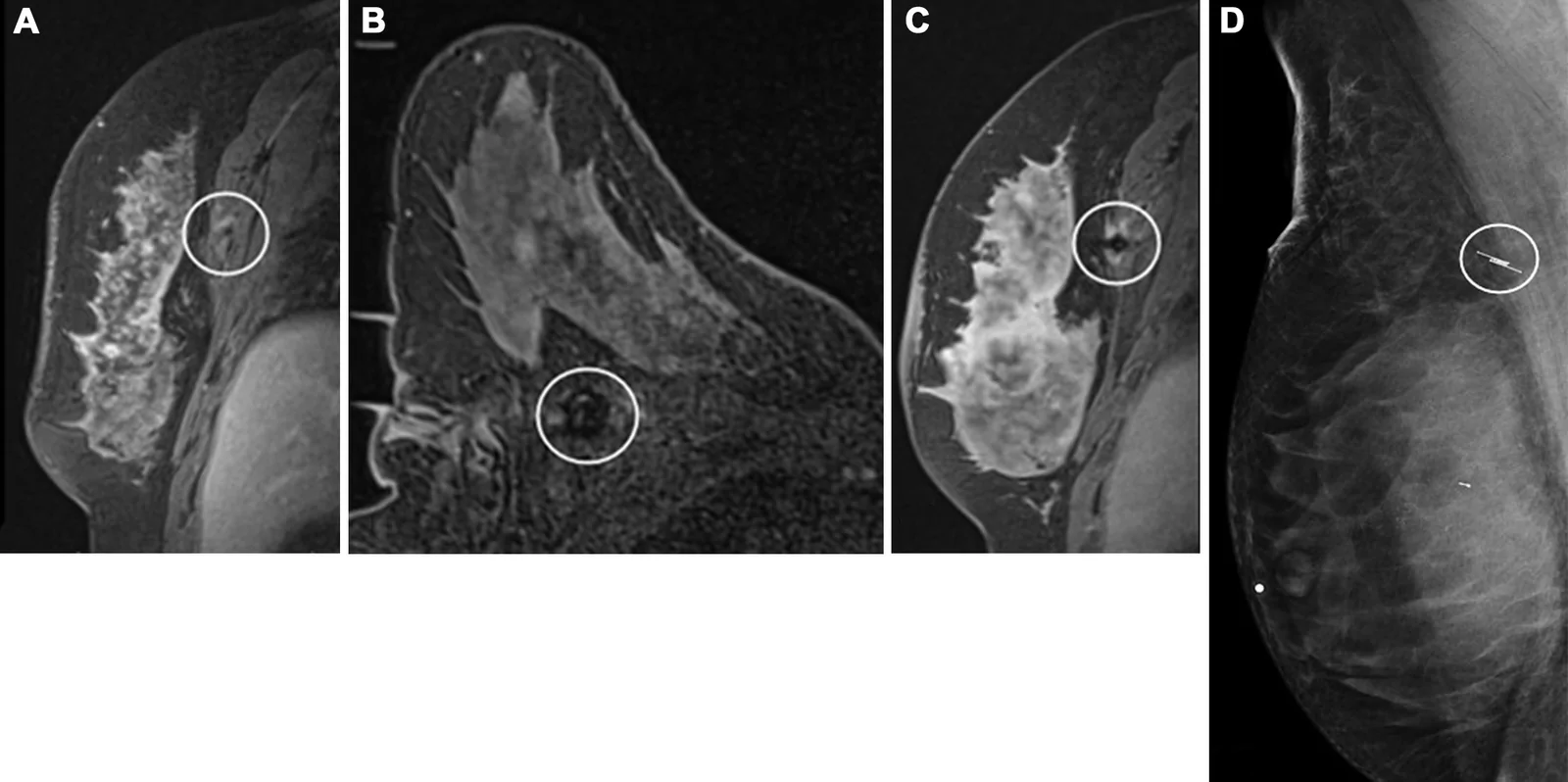
Savi Scout localization of residual pectoralis muscle enhancement in a 28-year-old woman scheduled for total right mastectomy and axillary dissection. The Savi Scout system was chosen for localization due to its minimal MRI artifacts, allowing continued imaging surveillance. **(A)** Sagittal prelocalization MR image shows residual enhancement in the pectoralis muscle (circle). **(B)** Axial postlocalization MR image shows use of the Savi Scout system with minimal artifacts (circle). **(C)** Sagittal postlocalization MR image shows the Savi Scout positioned centrally in the residual pectoralis muscle enhancement (circle) with minimal imaging artifacts. **(D)** Postprocedural lateromedial mammogram shows adequate placement of the Savi Scout system (circle). The radar-based Savi Scout system provides effective localization while preserving image quality for future MRI follow-up.

MRI compatibility and artifacts represent critical considerations for device selection, particularly in patients requiring neoadjuvant therapy monitoring. Savi Scout produces minimal signal void artifacts, whereas Magseed creates 2–6-cm signal voids and localizer RFID tags produce 2–8-cm artifacts (64,65). This difference is clinically significant, as Savi Scout’s minimal MRI artifacts do not interfere with imaging studies, allowing unrestricted use of MRI throughout treatment (Fig 11). In contrast, Magseed’s 4–6-cm signal void artifacts due to iron content may limit diagnostic accuracy of breast MRI, precluding placement in patients undergoing neoadjuvant chemotherapy when presurgical MRI reevaluation is required (66). Similarly, localizer systems produce signal intensity voids up to 8 cm (65). These imaging considerations make Savi Scout the preferred option for patients requiring MRI monitoring during neoadjuvant therapy, while Magseed and localizer systems are better suited for patients proceeding directly to surgery without intervening imaging requirements.

## Future Directions and Clinical Implications

The evolution of breast biopsy markers from simple metallic clips to sophisticated multimaterial devices reflects the broader advancement of precision medicine in breast care. From initial development at MD Anderson Cancer Center in the 1960s through FDA approval in 1995 and subsequent technological refinements, these devices have become indispensable tools supporting diagnostic accuracy and therapeutic precision.

Current clinical evidence strongly supports marker use in multiple domains: reliable imaging correlation, precise surgical planning, accurate radiation therapy targeting, and substantial cost savings through improved procedural efficiency. Although challenges including migration, allergic reactions, and visibility limitations persist, ongoing technologic advances continue to address these concerns through improved designs, alternative materials, and innovative detection methods such as Doppler US twinkling.

The integration of markers into routine clinical practice has fundamentally improved patient outcomes by ensuring accurate lesion localization, reducing repeat procedures, and maintaining care continuity across health care settings. As breast imaging technology continues to advance, biopsy markers will undoubtedly remain cornerstones of comprehensive breast care, supporting both diagnostic accuracy and therapeutic precision in breast disease management.

The future of breast biopsy markers lies in continued technologic innovation addressing current limitations while maintaining the proven benefits that have made these devices essential components of modern breast care. Through thoughtful selection, proper placement techniques, and understanding of marker-specific characteristics, clinicians can optimize patient outcomes while minimizing complications and ensuring seamless care coordination throughout the treatment continuum.

## Supplemental Files

Table S1

Conflicts of Interest
