# Supplementary material for: Selection of Breast Biopsy Markers: Effect on Breast Imaging Procedures, Follow-up, and Costs
Source: Radiographics. 2026 Aug 13;46(9):e250151. doi: 10.1148/rg.250151 (PMC13550975; doi:10.1148/rg.250151)
Supplement: Supplementary file 1 [file rg250151supp.pdf]

Supplemental materials undergo peer review but are not copyedited, and may include typographical errors.
©RSNA, 2026 • 10.1148/rg.250151

Table S1. Breast biopsy markers. This table provides detailed specifications including vendor information, brand names, material composition, and embedding materials for each marker type. Each marker is depicted through original artwork illustrating its distinctive morphology, pre-deployment characteristics, and post-deployment appearance within breast tissue. The information presented does not constitute endorsement by the contributing authors or their institutions. (All drawings are the original artwork of Kelly M. Kage, BS, MFA, CMI.)

Marker Name	Description				Shape		
	Vendor	Brand	Material	Embedding Material			
Anchor	Mammotome	(No longer commercially available)	Stainless Steel	None	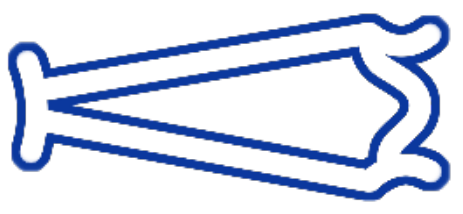	Historical reference only	Historical reference only
Barbell	Mammotome	MammoSTAR/ BiomarC	Ceramic/ Carbon	β-glucan	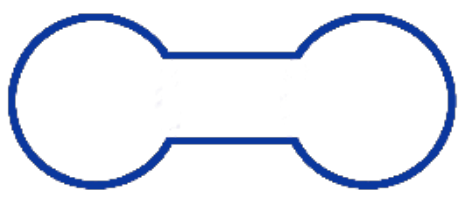	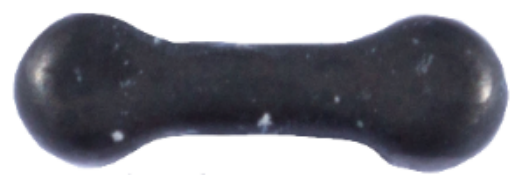	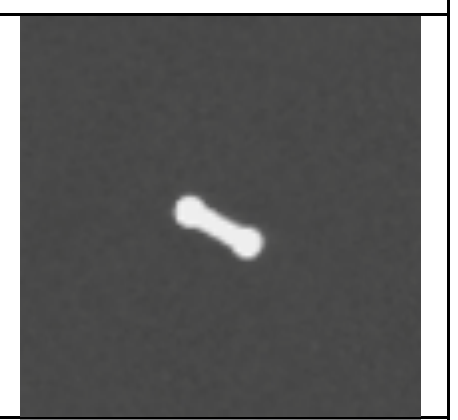
Barrel (T1)	Mammotome	HydroMARK	Titanium/ Stainless Steel	Hydrogel (PEG)	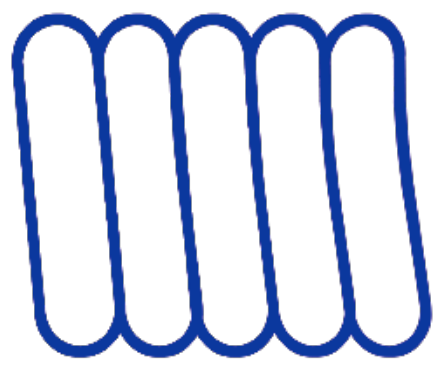	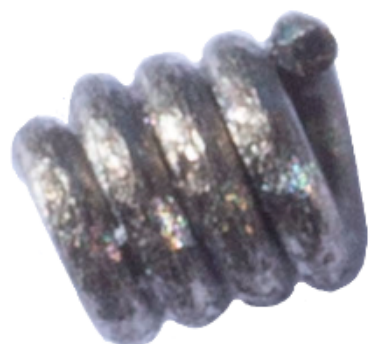	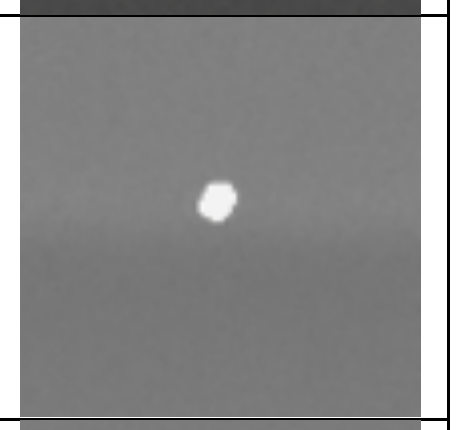
Beacon	Mermaid Medical	Mermaid	PEKK	None	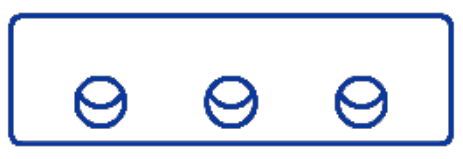	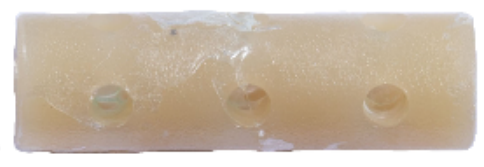	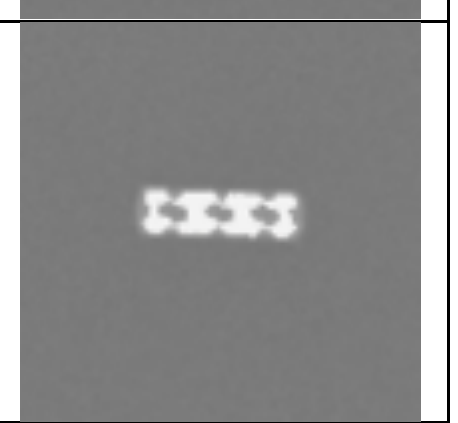
Bowtie	Mammotome	MammoMARK	Titanium	Collagen	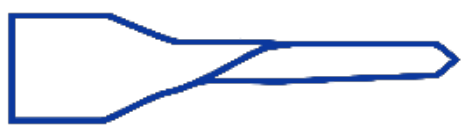	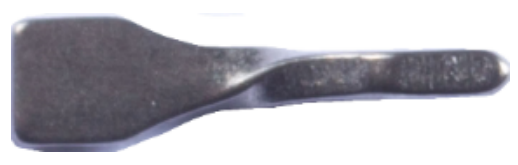	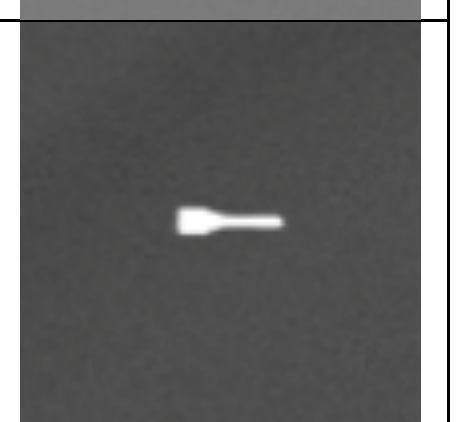
Buckle	Hologic	SecurMark	Stainless Steel	Bioabsorbable suture-like net	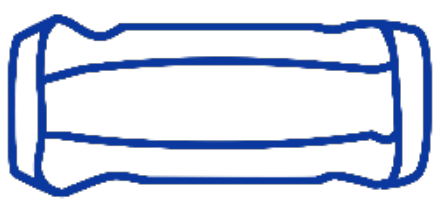	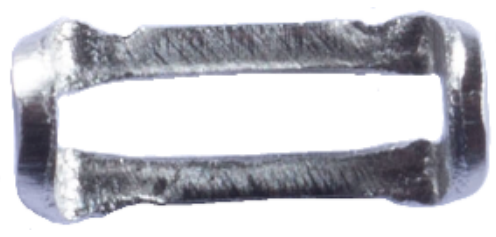	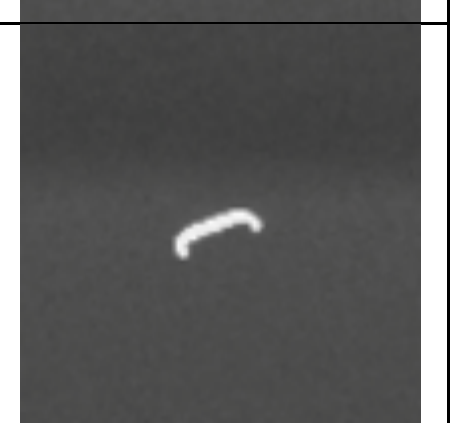
Butterfly (T4)	Mammotome	HydroMARK	Titanium/ Stainless Steel	Hydrogel (PEG)	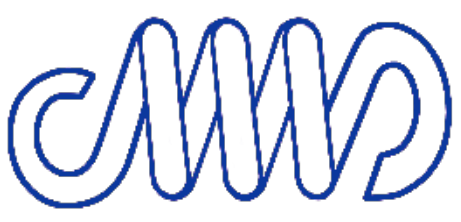	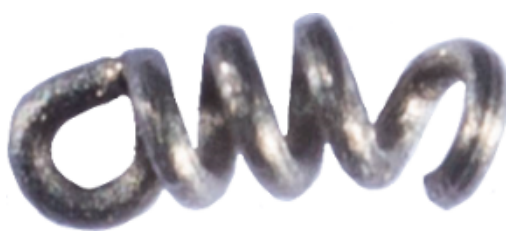	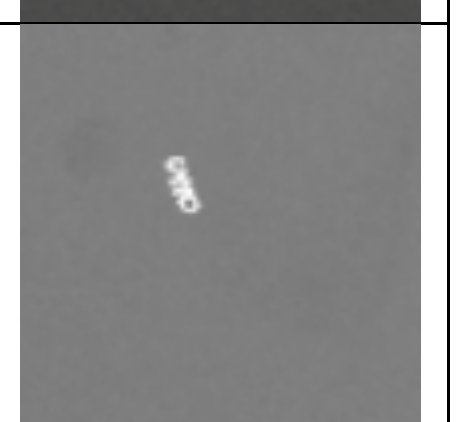
Cassi Star	Mermaid Medical	Mermaid	PEKK	None	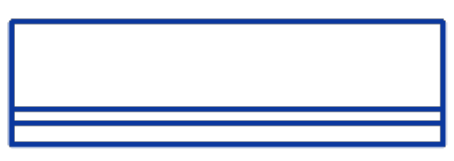	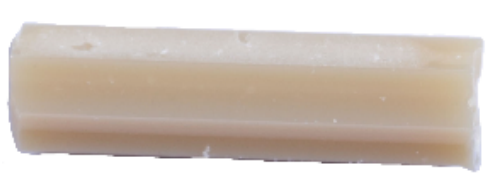	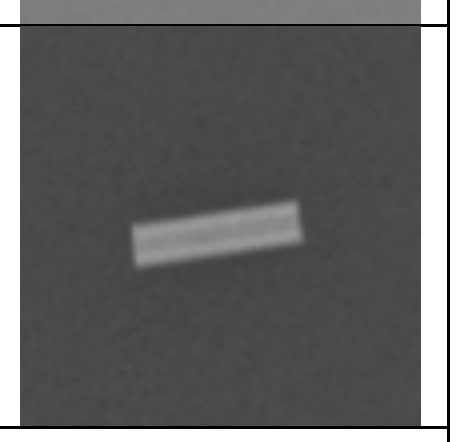
Clover	BD	UltraCOR™ Twirl™	Nitinol	None	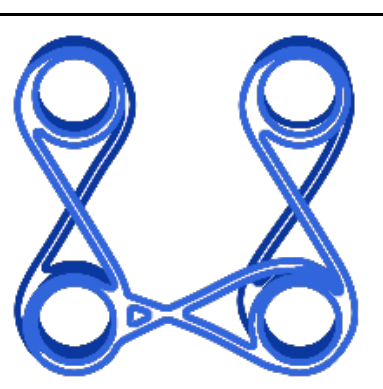	(See BD website)	
Coil	BD	SenoMark UltraCor	BioDur 108	PVA polymer and PGA microfiber pads	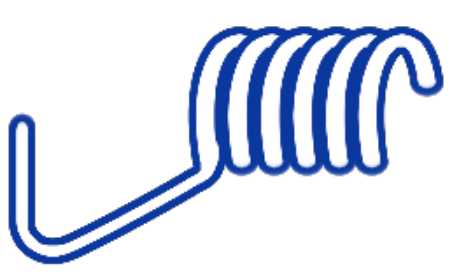	(See BD website)	
Conic	Hologic		Nitinol	None	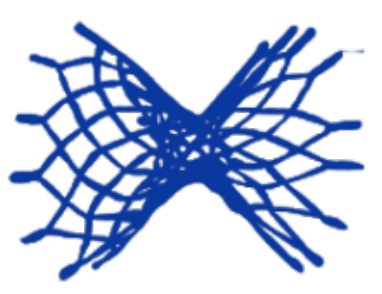	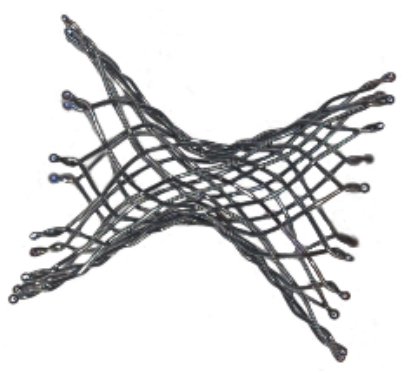	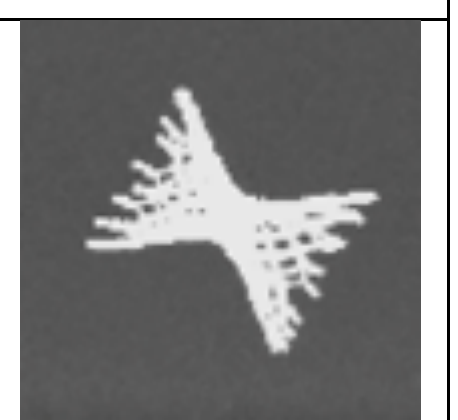
Cork	Hologic	TriMark	Titanium	None	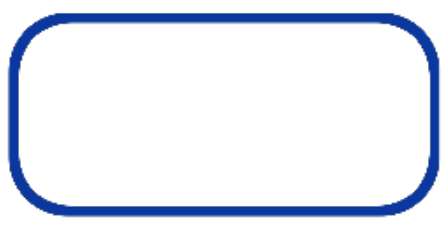	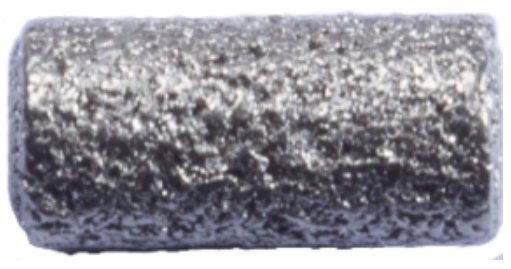	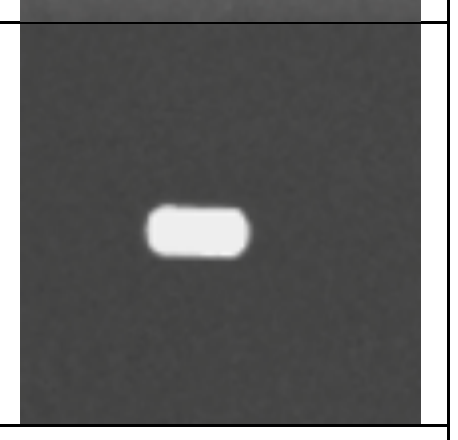
Curls	BD	UltraCOR™ Twirl™	Nitinol	None	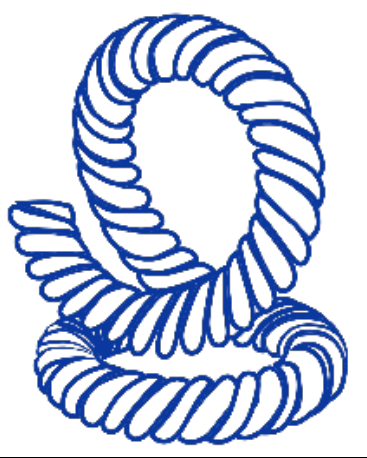	(see BD website)	

Supplemental materials undergo peer review but are not copyedited, and may include typographical errors.
©RSNA, 2026 • 10.1148/rg.250151

Dragonfly	Mammotome	HydroMARK Plus	Titanium	Hydrogel (PEG) partial embedding	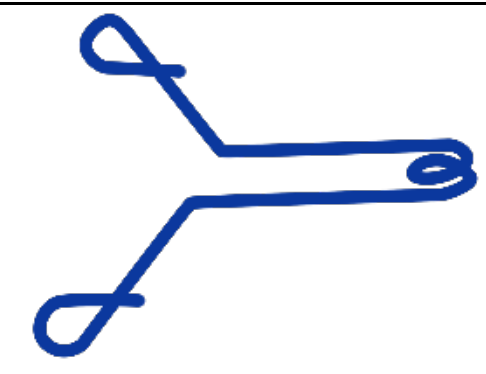	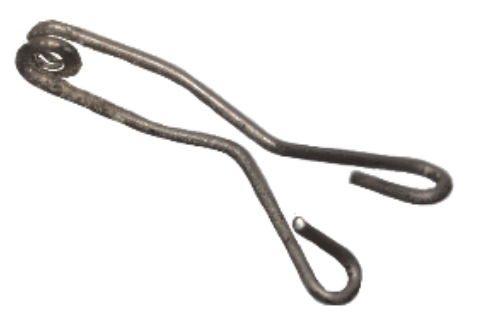	
Eye	Hologic	Tumark	Nitinol	None	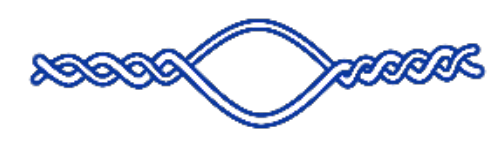	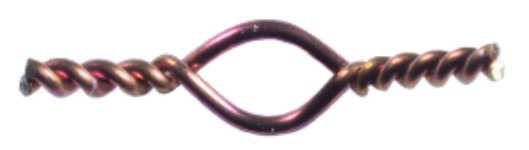	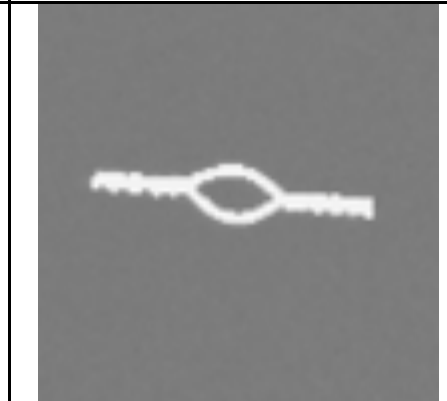
Flex	Hologic	Tumark	Nitinol	None	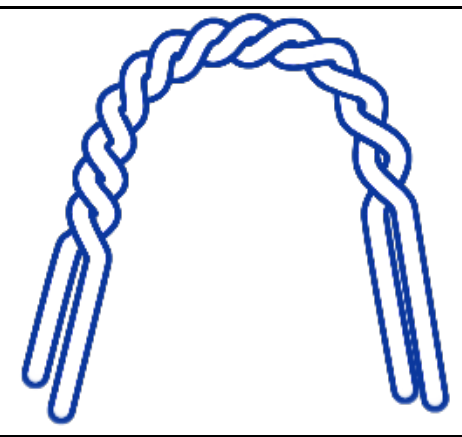	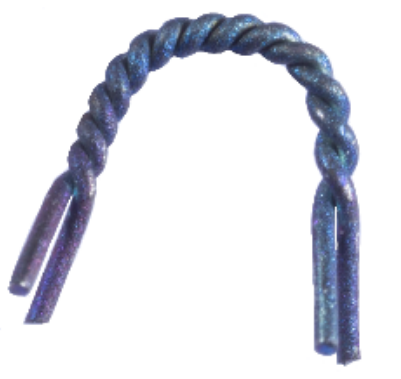	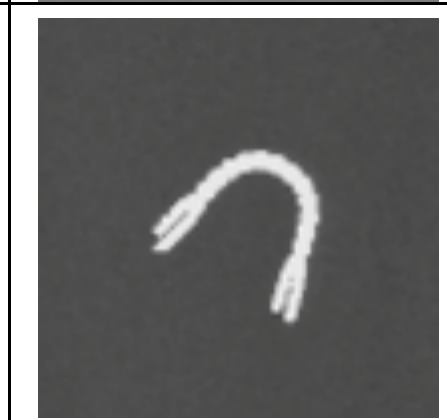
Heart	BD	SenoMark UltraCor	Titanium	PGA microfiber pad	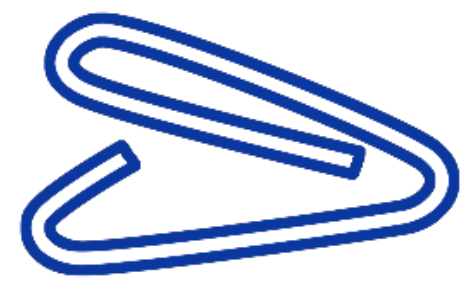	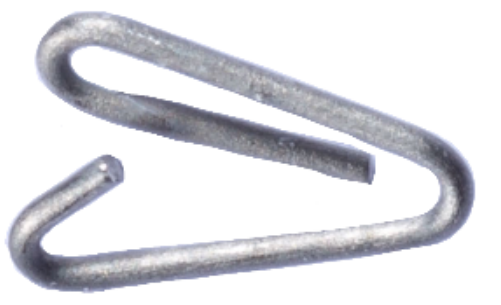	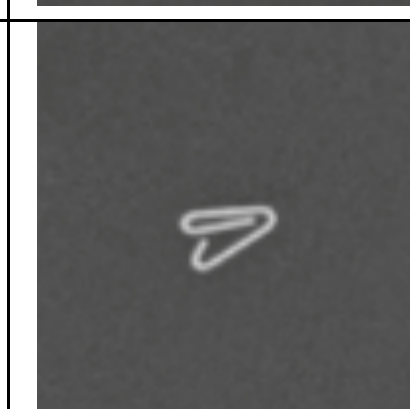
Hourglass	Hologic	TriMark	Titanium	None	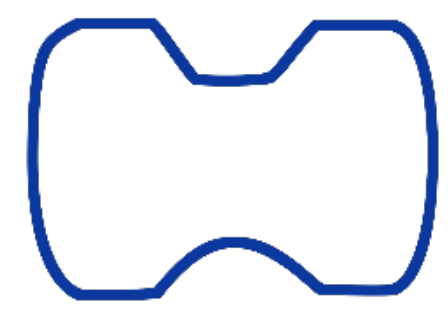	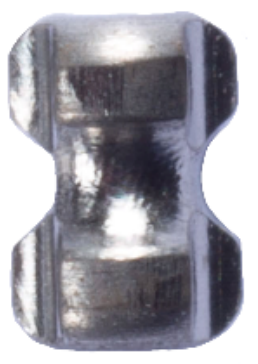	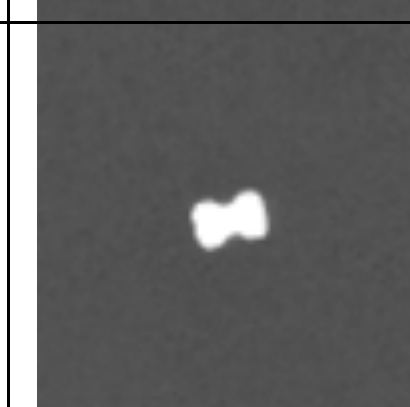
Hummingbird	Mammotome	HydroMARK Plus	Titanium	Hydrogel (PEG) partial embedding	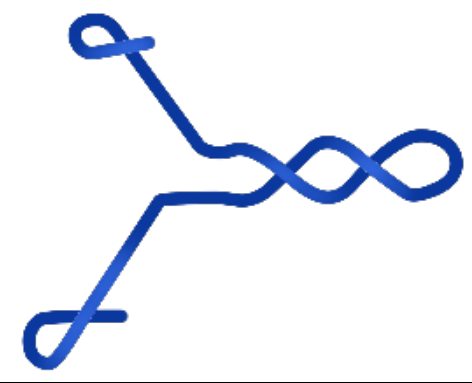	(see Mammotome website)	
Infinity	Hologic	SecurMark	Stainless Steel	Bioabsorbable suture-like net	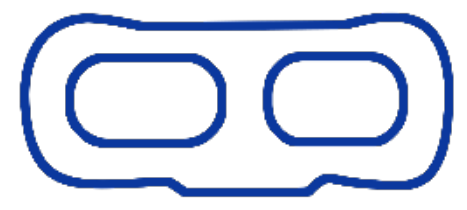	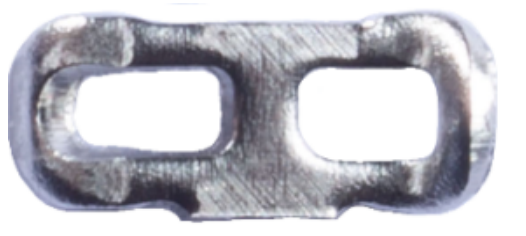	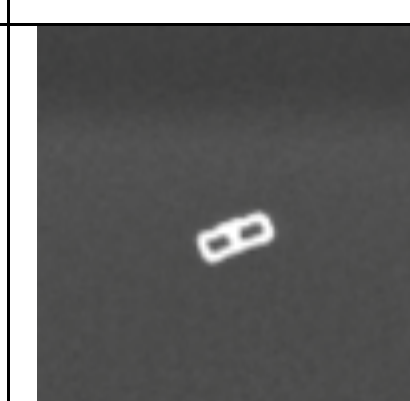
Lotus	Mammotome	LumiMARK	Nitinol	None	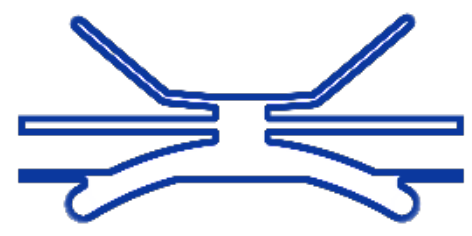	(see Mammotome website)	
M	BD	SenoMark	315L Stainless Steel	PGA microfiber pads	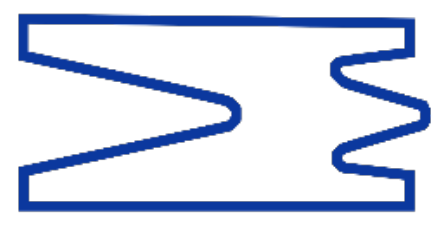	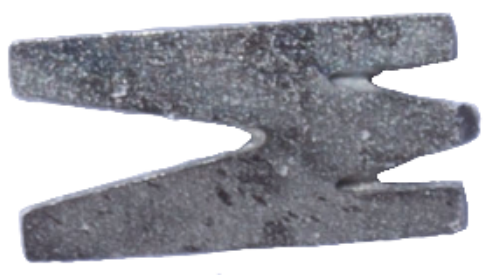	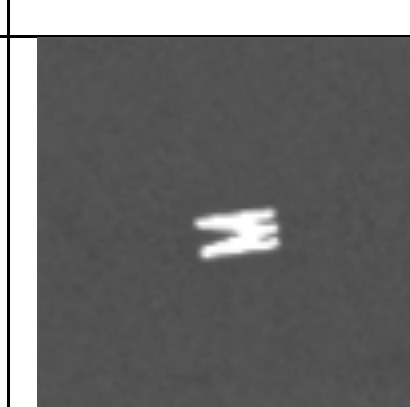
Mini-Cork	Hologic	SecurMark	Titanium	Bioabsorbable suture-like net	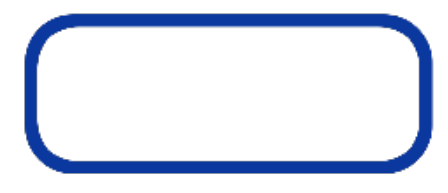	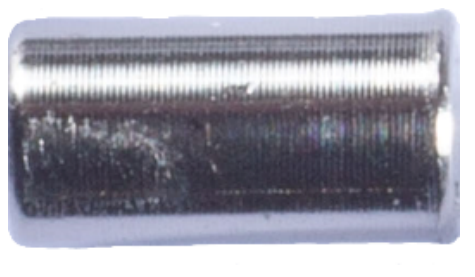	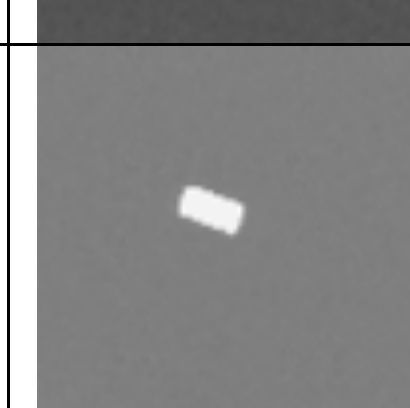
MRI (Flex)	Somatex	Tumark	Nitinol	None	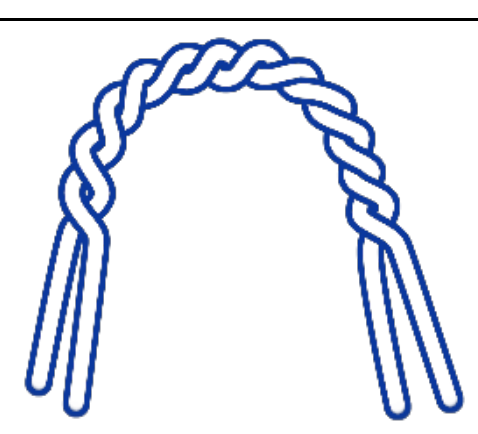	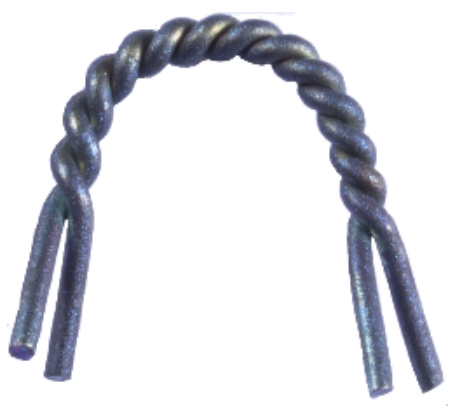	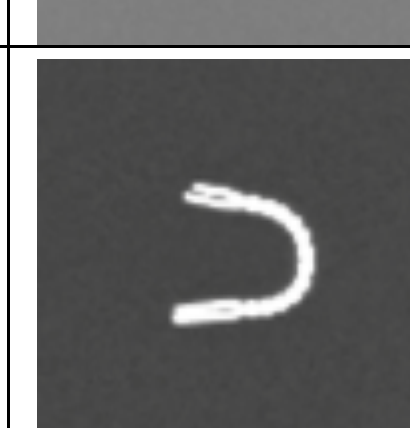
O	BD	SenoMark	Titanium	PGA microfiber pads	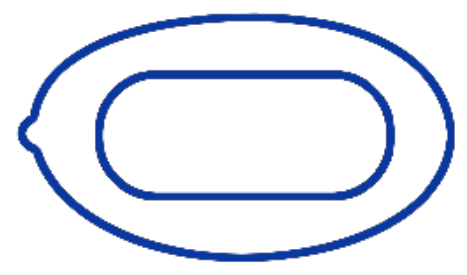	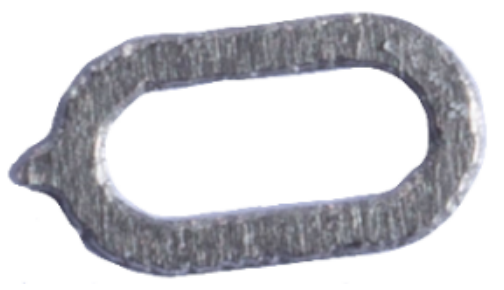	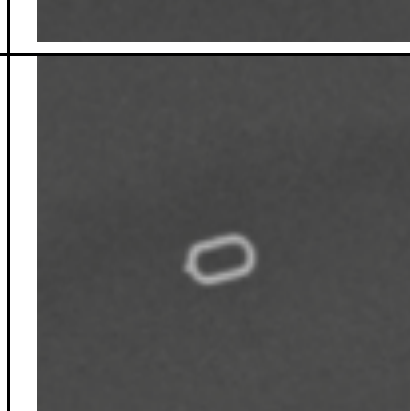
Omega	BD	SenoMark	Stainless Steel	PGA microfiber pads	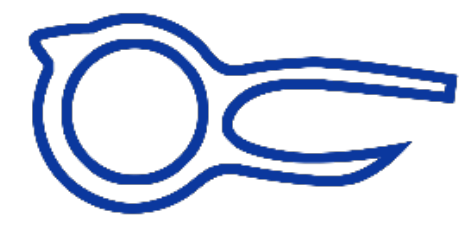	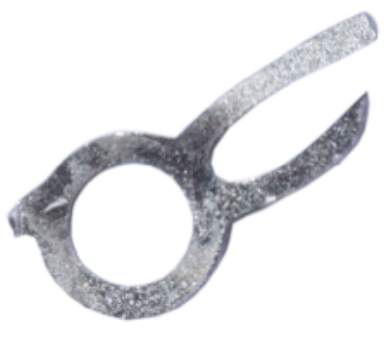	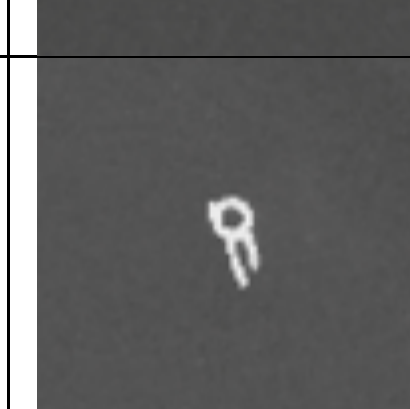
Open Coil (T3)	Mammotome	HydroMARK	Titanium/ Stainless Steel	Hydrogel (PEG)	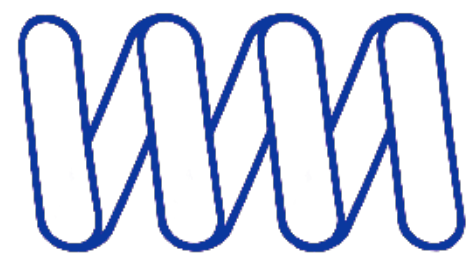	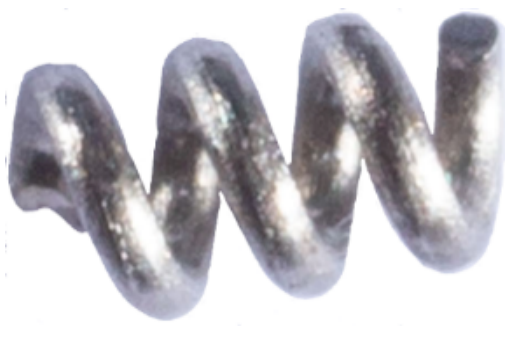	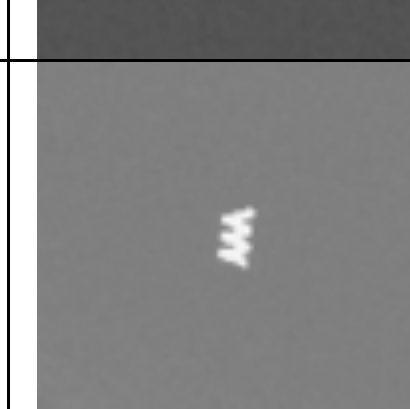

Supplemental materials undergo peer review but are not copyedited, and may include typographical errors.
©RSNA, 2026 • 10.1148/rg.250151

Professional Q	Hologic	Tumark	Nitinol	None	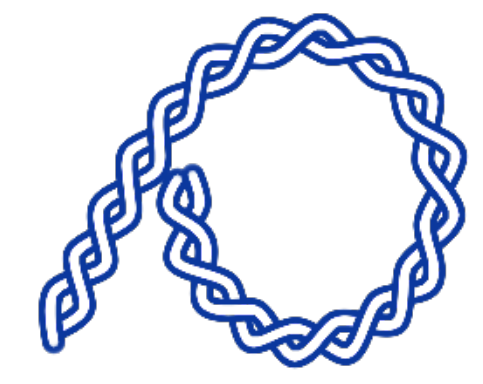	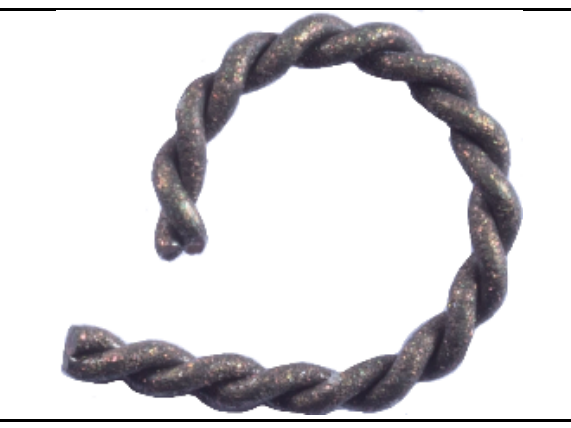	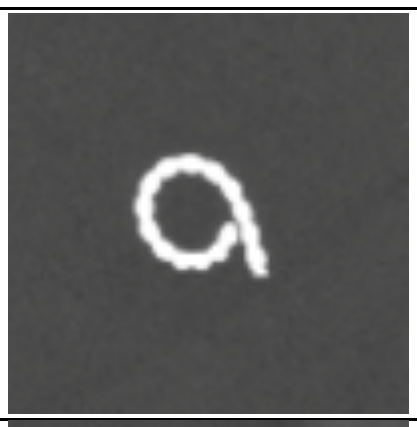
Professional X	Hologic	Tumark	Nitinol	None	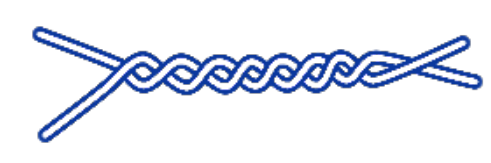	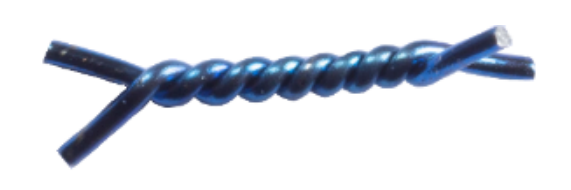	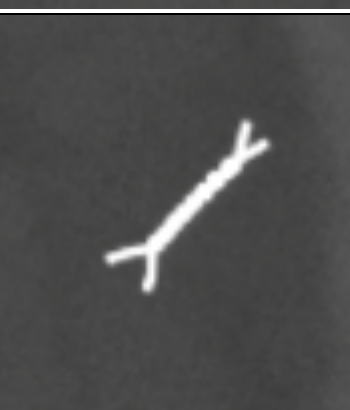
Ribbon	BD	UltraClip	Titanium	Interwoven PVA polymer	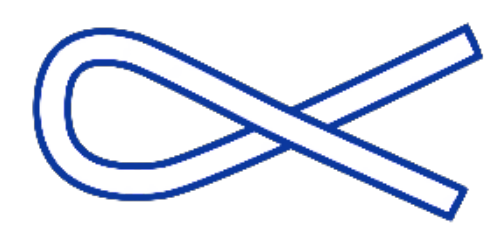	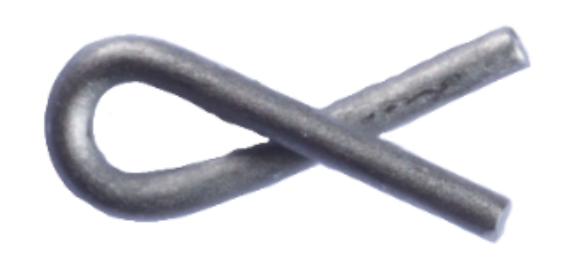	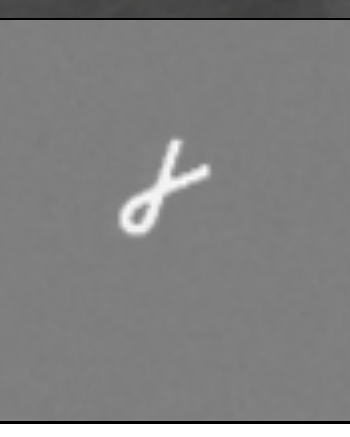
Ring	BD	UltraCor™ Twirl™	Nitinol	None	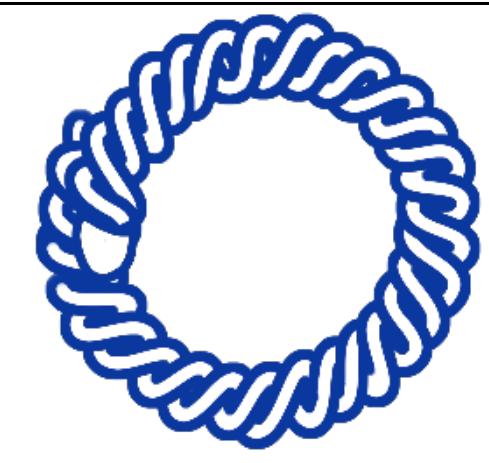	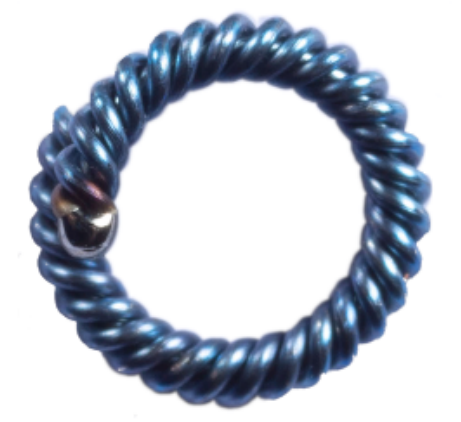	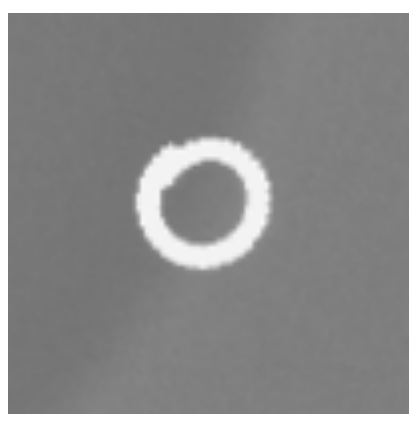
Rose	Mammotome	LumiMARK	Nitinol	None	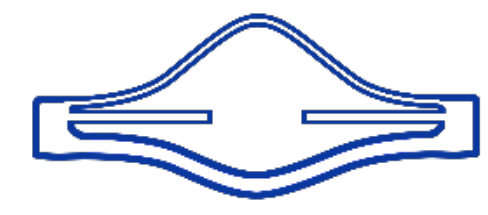	(see Mammotome website)	
S	BD	SenoMark	Titanium	PGA microfiber pads	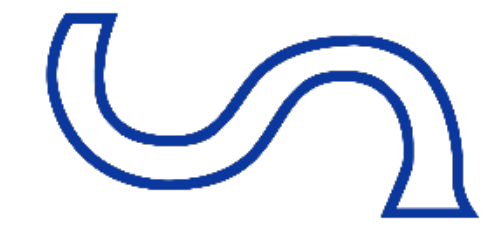	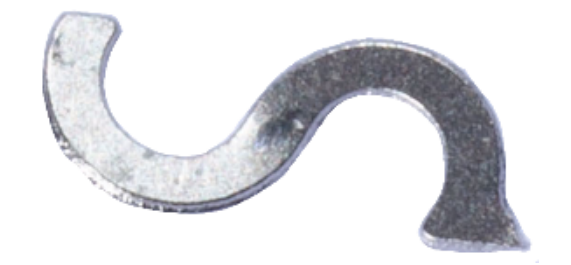	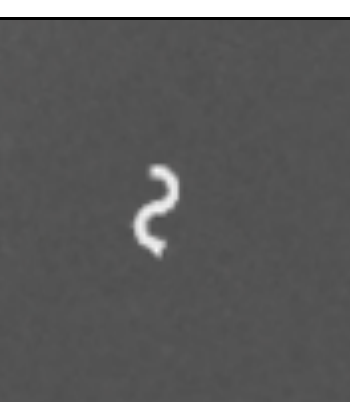
Spring	BD	UltraCor	316L Stainless Steel	PEG plugs	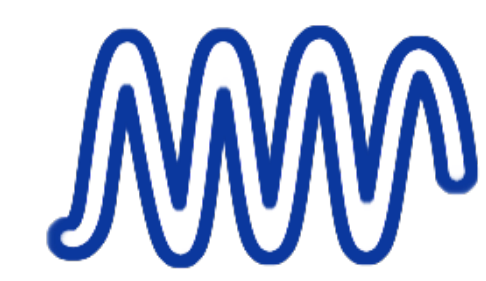	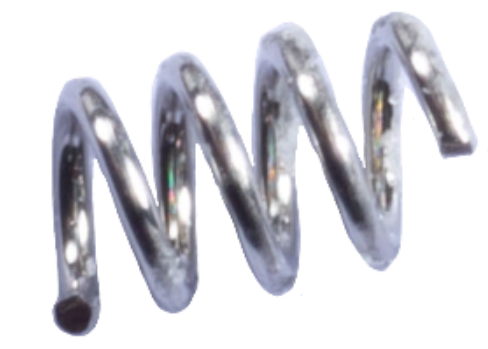	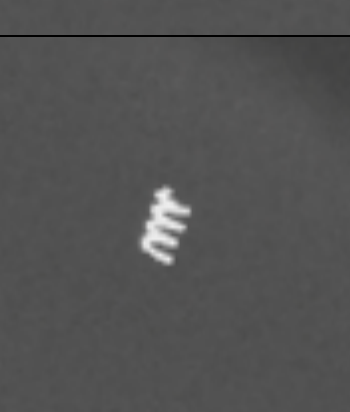
Stoplight	Hologic	SecurMark	Stainless Steel	Bioabsorbable suture-like net	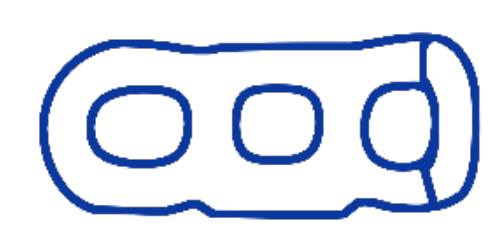	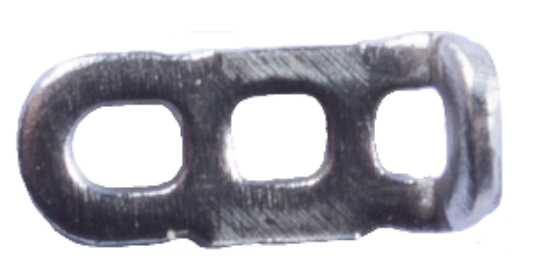	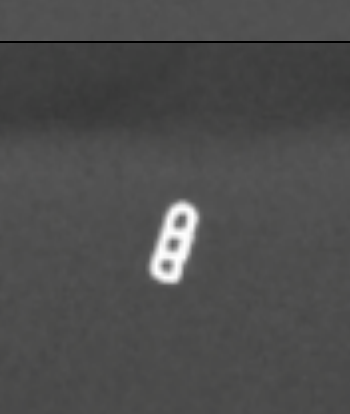
Tophat	Hologic	SecurMark	Titanium	Bioabsorbable suture-like net	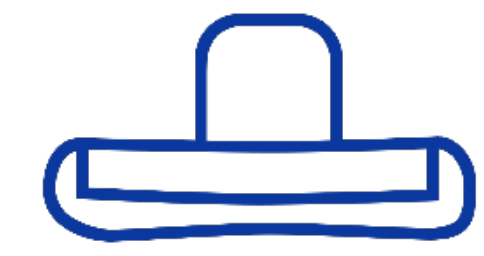	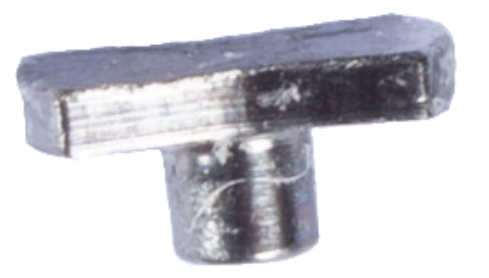	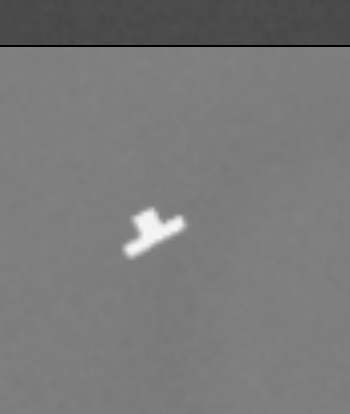
Tribell	Mammotome	MammoSTAR/ BiomarC	Ceramic/ Carbon	β-glucan	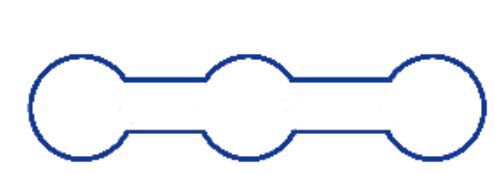	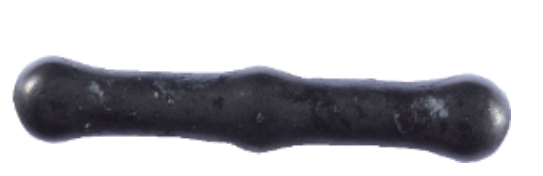	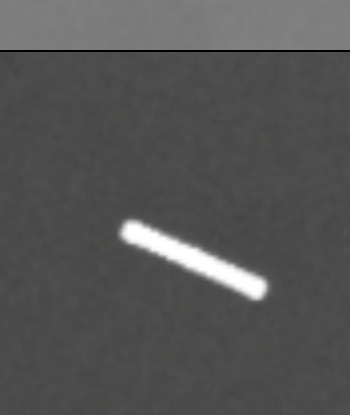
Triple Twist	Mammotome	MammoMARK	Titanium	Collagen	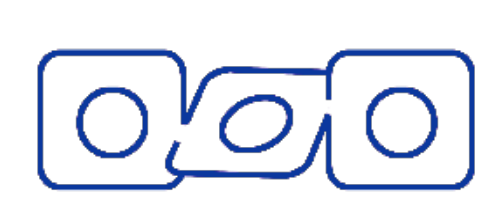	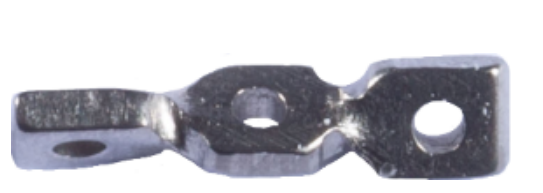	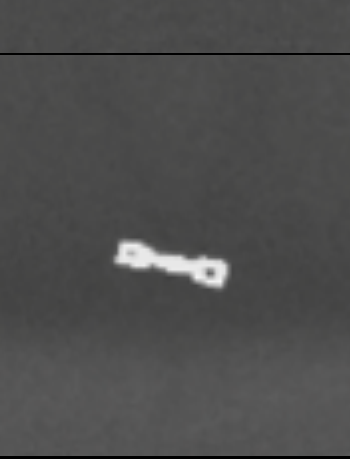
Tulip™	Mammotome	LumiMARK	Nitinol	None	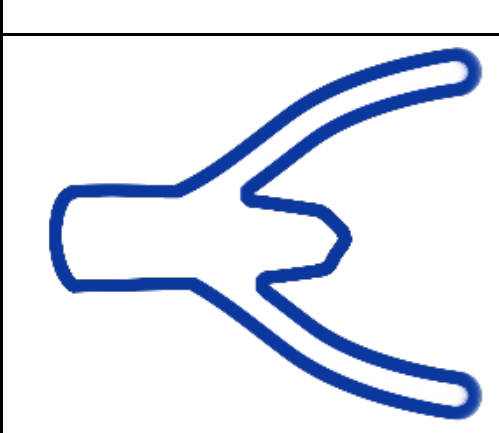	(see Mammotome website)	
U	Mammotome	MammoMARK	Titanium	Collagen	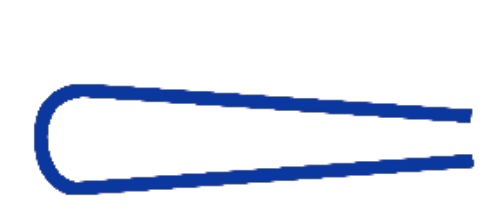	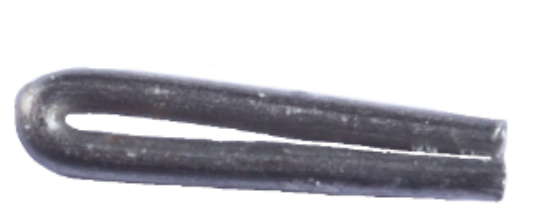	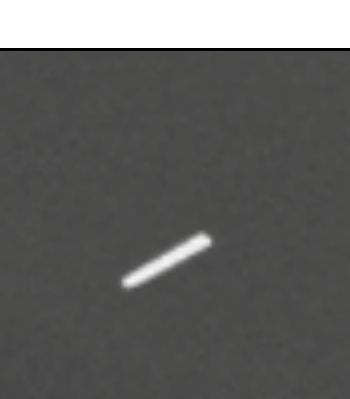
V (no longer commercially available)	BD	StarchMark UltraCor	316L Stainless Steel	Starch pellets	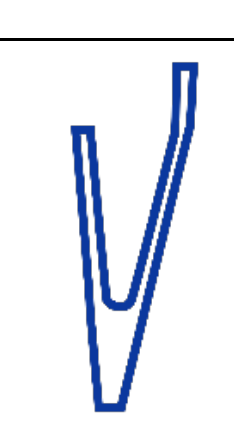	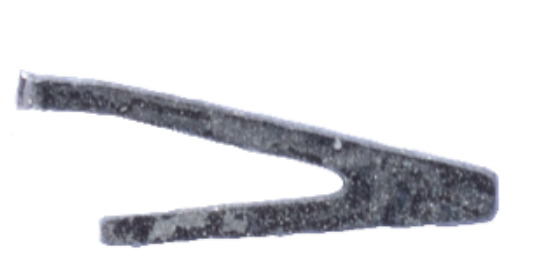	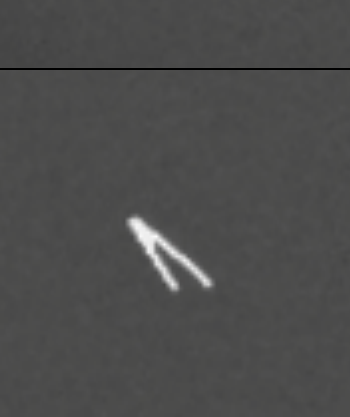

Supplemental materials undergo peer review but are not copyedited, and may include typographical errors.
©RSNA, 2026 • 10.1148/rg.250151

Venus	BD	SenoMark UltraCor	BioDur 108	PGA microfiber pad	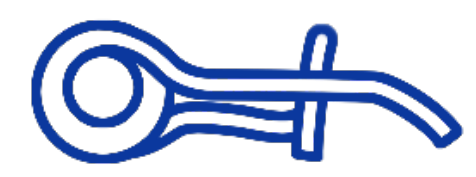	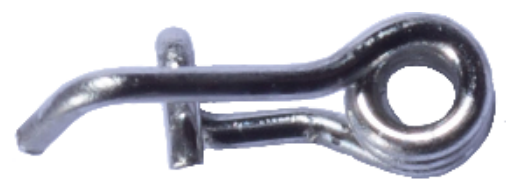	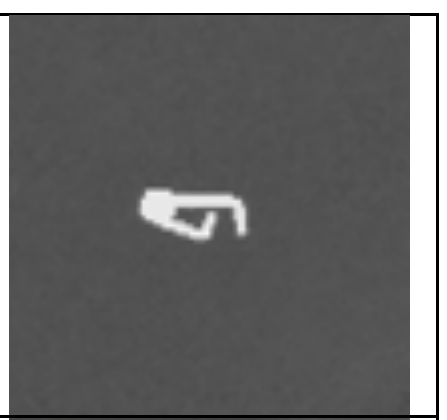
Vision	Hologic	Tumark	Nitinol	None	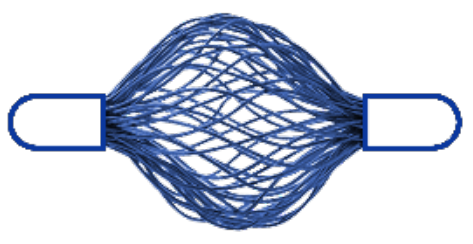	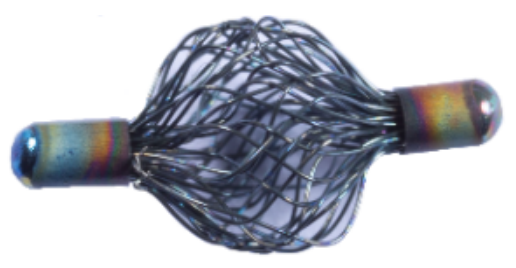	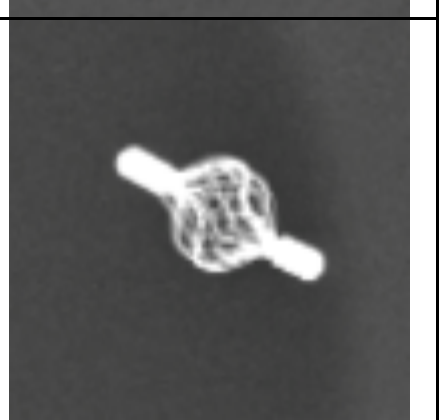
Wing	BD	UltraClip II	Iconel™ 625	None	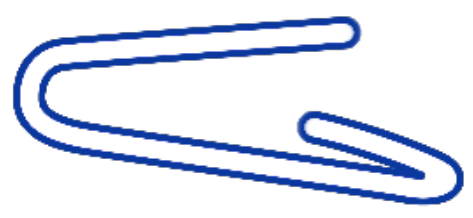	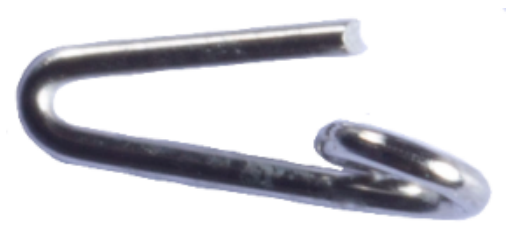	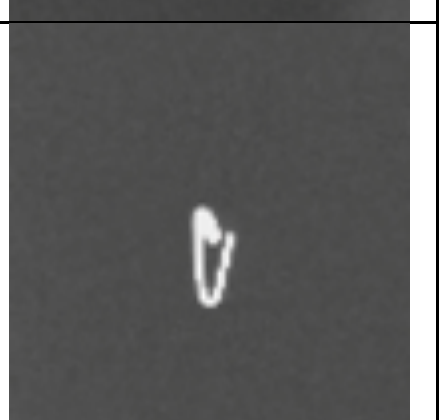
X	BD	SenoMark UltraCor MRI	Titanium	PGA microfiber pads	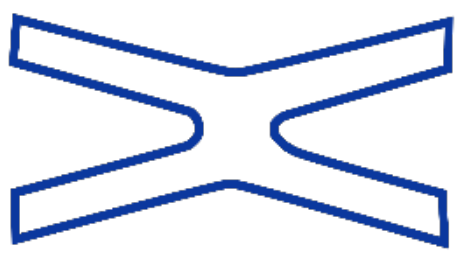	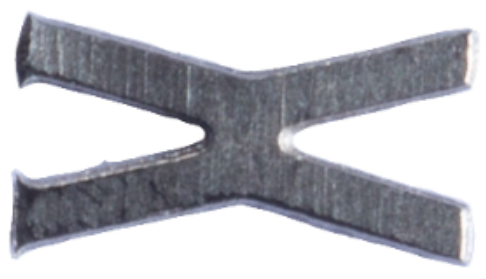	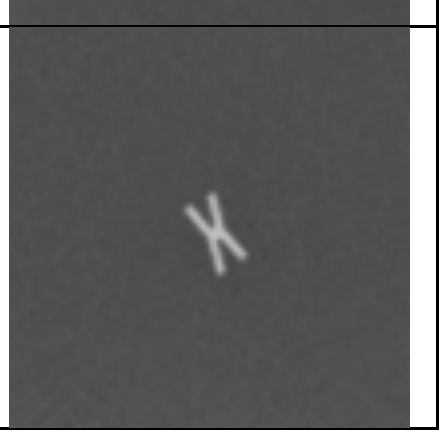
